# Close and distant: Contrasting the metabolism of two closely related subspecies of Scots pine under the effects of folivory and summer drought

**DOI:** 10.1002/ece3.3343

**Published:** 2017-09-25

**Authors:** Albert Rivas‐Ubach, Jordi Sardans, José Antonio Hódar, Joan Garcia‐Porta, Alex Guenther, Ljiljana Paša‐Tolić, Michal Oravec, Otmar Urban, Josep Peñuelas

**Affiliations:** ^1^ Environmental Molecular Sciences Division Pacific Northwest National Laboratory Richland WA USA; ^2^ CREAF Bellaterra, Barcelona Spain; ^3^ CSIC Global Ecology Unit CREAF‐ CSIC‐UAB Bellaterra, Barcelona Spain; ^4^ Grupo de Ecología Terrestre Departamento de Biología Animal y Ecología Facultad de Ciencias Universidad de Granada Granada Spain; ^5^ Department of Earth System Science University of California Irvine CA USA; ^6^ Global Change Research Institute Czech Academy of Sciences Bělidla 986/4a 603 00 Brno Czech Republic

**Keywords:** drought, evolutionary processes, folivory, herbivorous attack, metabolomics, *Pinus sylvestris*, processionary moth, sympatric subspecies

## Abstract

Metabolomes, as chemical phenotypes of organisms, are likely not only shaped by the environment but also by common ancestry. If this is the case, we expect that closely related species of pines will tend to reach similar metabolomic solutions to the same environmental stressors. We examined the metabolomes of two sympatric subspecies of *Pinus sylvestris* in Sierra Nevada (southern Iberian Peninsula), in summer and winter and exposed to folivory by the pine processionary moth. The overall metabolomes differed between the subspecies but both tended to respond more similarly to folivory. The metabolomes of the subspecies were more dissimilar in summer than in winter, and *iberica* trees had higher concentrations of metabolites directly related to drought stress. Our results are consistent with the notion that certain plant metabolic responses associated with folivory have been phylogenetically conserved. The larger divergence between subspecies metabolomes in summer is likely due to the warmer and drier conditions that the northern iberica subspecies experience in Sierra Nevada. Our results provide crucial insights into how *iberica* populations would respond to the predicted conditions of climate change under an increased defoliation in the Mediterranean Basin.

## 1 INTRODUCTION

An organism's metabolome consists of thousands of compounds of low molecular weight (metabolites) present in an organism at a given time (Fiehn, 2002). Such molecules include the substrates and products of cellular primary metabolism, such as sugars, amino acids, and nucleotides, and of secondary metabolism that are involved in a large variety of complex physiological processes for maintaining homeostasis and normal function. The metabolome is the chemical phenotype of an organism (Fiehn, 2002) and is the first to respond to biotic and abiotic stressors (Peñuelas & Sardans, 2009). The recent application of new metabolomic techniques in the fields of plant physiology and ecology (ecometabolomics) has allowed the detection of the extreme plasticity of metabolomes under different environmental situations (Rivas‐Ubach, Sardans, Pérez‐Trujillo, Estiarte, & Peñuelas, 2012; Rivas‐Ubach, Barbeta, et al., 2016; Sardans, Peñuelas, & Rivas‐Ubach, 2011). However, the metabolome, as any other aspect of the phenotype, can also be subject to evolutionary divergence given that metabolic responses ultimately depend on genetic composition and expression (Riedl et al., 2012). From this point of view, we expect that closely related organisms will have more similar metabolomes than distantly related organisms. If this is the case, environmental changes would not necessarily lead to a complete reorganization of an organism's metabolome, because genetic and evolutionary constraints would determine several metabolomic characteristics. Therefore, closely related species exposed to the same environmental conditions would reach similar metabolic solutions in response to similar environmental changes, including abiotic stressors (such as drought), abiotic fluctuations (such as seasonal variability), and biotic stressors (such as herbivore pressure).

Understanding the environmental and phylogenetic contributions of the metabolome is critical in the current context of global environmental change (Edwards, Still, & Donoghue, 2007; González‐Orozco et al., 2016; Kuntner, Năpăruş, Li, & Coddington, 2014). Ecosystems are currently facing an environmental change of planetary dimensions, including a global increase in average temperatures and some areas with substantial increases in aridity, such as the Mediterranean Basin (IPCC, 2002). Aside from these abiotic effects, climate change also produces increases in the virulence of pest attacks in certain areas of the planet (Battisti et al., 2015). Organisms will have to respond to all these changes, particularly plants that do not have the capability to drastically change altitudinal or geographical distributions over short timescales (Chen, Hill, Ohlemüller, Roy, & Thomas, 2011; Gonzalez, Neilson, Lenihan, & Drapek, 2010; Meier, Lischke, Schmatz, & Zimmermann, 2012; Parmesan & Yohe, 2003). Different phylogenetic and environmental contributions to the metabolome can potentially induce different sensitivities to climate change in different plant species, and therefore, studies focusing on this issue are critical to understanding how different species and ecosystems will respond to this global threat.

**Figure 1:**
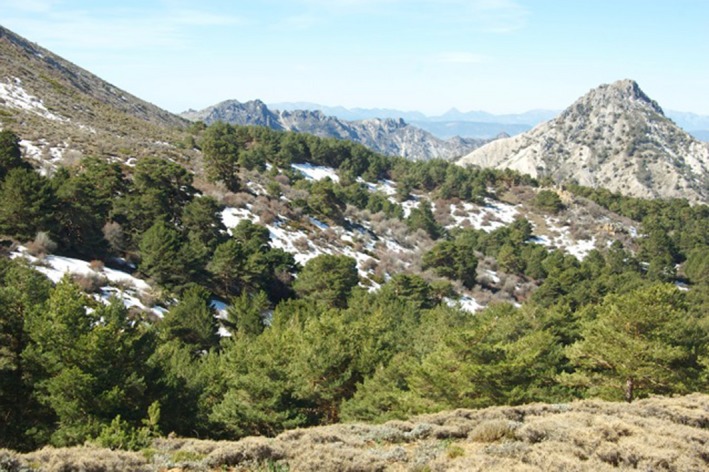
Scots pine forests of Collado de Matasverdes in Sierra Nevada National Park. The photograph illustrates part of the study site where *Pinus sylvestris* ssp. *nevadensis* (native) coexists with *P. sylvestris* ssp. *iberica* (introduced). Photograph by Dr. José Antonio Hódar

**Figure 2:**
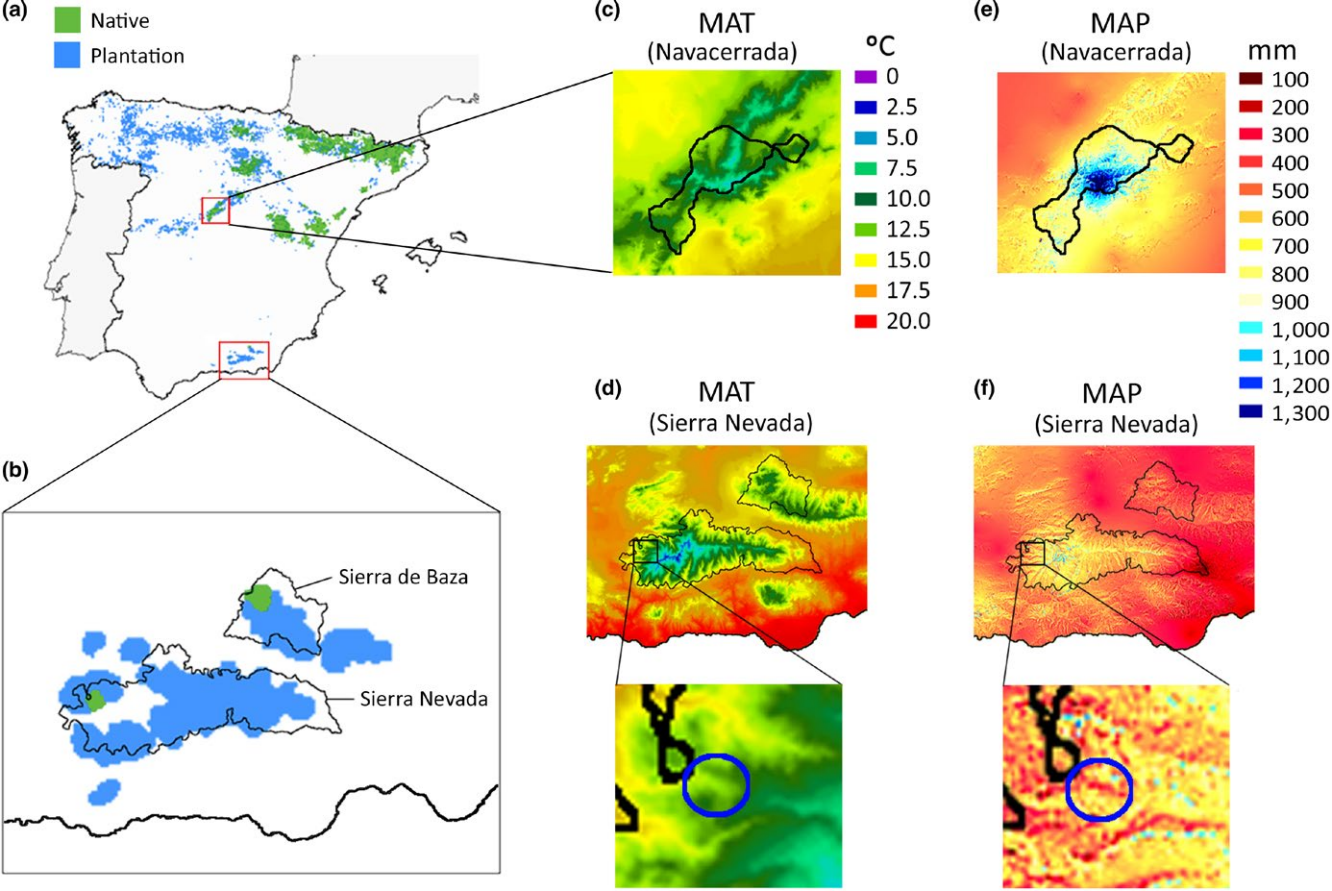
Native (green) and planted (blue) populations of Scots pine across Spain (a) and in Sierra Nevada and Sierra de Baza (b). Native populations in Sierra Nevada and Sierra de Baza are *Pinus sylvestris* ssp. *nevadensis*. Maps of mean annual temperature (MAT) for Navacerrada, the native habitat of *P. sylvestris* ssp. *iberica* planted in southern Spain, (c) and for Sierra Nevada, the study site, (d) and of mean annual precipitation (MAP) for Navacerrada (e) and Sierra Nevada (f) are provided. Native populations of *P. sylvestris* ssp. *iberica* in Navacerrada are delimited by the black lines in (c) and (e). The study site is identified by the blue circles in the magnifications of Sierra Nevada in (d) and (f). Maps with the distribution of native and introduced *P. sylvestris* woodlands were kindly supported by the Spanish network on Genetics and Conservation of Forest Resources (GENFORED). Climatic maps were obtained from the “Atlas Climático Digital de la Península Ibérica” (Ninyerola, Pons Fernàndez, & Roure i Nolla, 2005)

**Figure 3:**
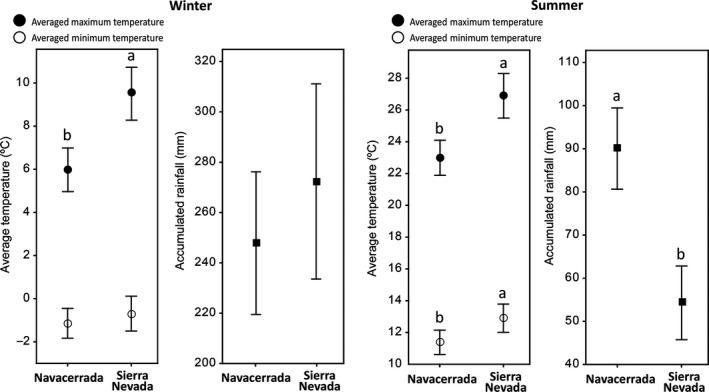
Average maximum (solid dots) and minimum (open dots) temperatures (°C) and accumulated rainfall (mm) (solid squares) for Navacerrada and Sierra Nevada Natural Park for winter and summer. The temperatures are means ± 3 *SE*. The accumulated rainfalls are means ± *SE*. Different letters denote significant differences between Navacerrada and Sierra Nevada (*p* < .05) identified by a *t* test (see Table S3)

We took advantage of a semi‐experimental situation where trees of a subspecies of Scots pine were transplanted from their natural habitat in central Spain to southern mountains. We explored whether the metabolomes of closely related species respond similarly to environmental conditions or stressors. Scots pine (*Pinus sylvestris* L.) is one of the most important and widespread forest trees in the Holarctic (Ceballos & Ruiz de la Torre, 1971; Gausen, Heywood, & Chater, 1964) and is an important species both ecologically and economically (Mäkinen & Hynynen, 2014). Natural *P. sylvestris* populations in Sierra Nevada Natural Park (southern Iberian Peninsula), described as *P. sylvestris* ssp. *nevadensis* (hereafter *nevadensis*) (Boratynski, 1991; Ceballos & Ruiz de la Torre, 1971; Gausen et al., 1964), were intensively thinned during the 19th century (Hódar, Castro, & Zamora, 2003) and represent the most southern populations of Scots pine. Recently, the endemic *nevadensis* populations are consequently protected for their ecological importance (Blanca, Cueto, Martínez‐Lirola, & Molero‐Mesa, 1998; Hódar et al., 2003), but environmental pressures such as defoliation by the pine processionary moth (hereafter PPM) *Thaumetopoea pityocampa* (Denis & Schiffermüller) are threatening their survival (Castro, Gómez, García, Zamora, & Hódar, 1999; Hódar & Zamora, 2004). Many areas originally covered by *nevadensis* were massively reforested later in the mid‐twentieth century with Scots pines from higher latitudes of Spain (Figure 1), specifically from the Sistema Central mountain range (Navacerrada, central Iberian Peninsula, 450 km north of Sierra Nevada) (Robledo‐Arnuncio, Navascués, González‐Martínez, & Gil, 2009), that belongs to another subspecies, *P. sylvestris* ssp. *iberica* (hereafter *iberica*). The *nevadensis* (native) and *iberica* (introduced) subspecies consequently coexist in some localities of Sierra Nevada and Sierra de Baza (Figure 2). These localities provide a unique opportunity to study the metabolomes of two tree subspecies under the same environmental conditions. This study thus allows a direct comparison between *nevadensis*, adapted to the southern conditions with higher temperature and lower rainfall in summer than Navacerrada (Figures 2 and 3), and the introduced *iberica* populations in Sierra Nevada, which may already be experiencing the environmental conditions projected for the near future (IPCC, 2002).

In this study, we specifically contrasted the metabolomes of *nevadensis* and *iberica* under the same environmental conditions of Sierra Nevada. In particular, our main aim was to determine whether the overall metabolomes of both subspecies tended to converge or diverge to two common stressors: (1) attack by PPM caterpillars, the main insect defoliator of *Pinus* species in the Mediterranean area (Battisti et al., 2015) and (2) the natural summer conditions of Sierra Nevada.

On the one hand, we expect that closely related sympatric species or subspecies will reach similar metabolomic solutions to both stressors due to their shared evolutionary history; however, many studies have reported a low degree of metabolomic conservation between plant species of the same genus exposed to drought (Sánchez, Schwabe, Erban, Udvardi, & Kopka, 2012) or herbivorous attack (Rivas‐Ubach, Hódar, et al., 2016), suggesting that metabolic responses to stressors could be taxon specific. To test these hypotheses in natural populations of trees, we sampled needles of both *P. sylvestris* subspecies in Sierra Nevada Natural Park in winter, the main period of PPM attack, and in summer, once the trees had flushed their needles after PPM attack. The foliar metabolomes of attacked trees (ATs) and nonattacked trees (NATs) of both subspecies were analyzed by liquid chromatography coupled to mass spectrometry (LC‐MS), and the obtained data were subsequently submitted to diverse univariate and multivariate statistical analyses to address our hypotheses. This study provides crucial information of the metabolomic flexibility of Scots pine to the attack by PPMs and to climate change, producing key knowledge for a better sustainable management of pine in a context of climate change.

## 2 MATERIAL AND METHODS

### 2.1 Study site

Foliar samples were collected in the *P. sylvestris* forests of Collado de Matasverdes (37.05°N, 3.27°W; 1,900 m a.s.l.) in Sierra Nevada National Park (Granada, SE Spain; Figure 2a,b,d,f), where *nevadensis* coexists with *iberica* in the same valley (Robledo‐Arnuncio et al., 2009) (Figures 1 and 2). Navacerrada, the native area of *iberica* trees (Figure 2a), has a mean annual temperature (MAT) of 6.4°C (Figure 2c) and a mean annual precipitation (MAP) of 1,330 mm (Figure 2e). The climate in Sierra Nevada is more Mediterranean, with hot summers, cold winters, and usually a severe summer drought. The MAT is 9.8°C (Figure 2d), and the MAP is 945 mm (Figure 2f). The minimum temperatures in winter and the maximum temperatures in summer, together with precipitation, are crucial factors determining the distributional niche of most plant species. The average minimum and maximum temperatures and the accumulated rainfalls for winter and summer for Navacerrada and the study site in Sierra Nevada are shown in Figure 3. Navacerrada climatic data were extracted by interpolating the data from the four stations closest to the *iberica* natural populations from the database AEMET (www.aemet.es). Sierra Nevada data were obtained from a meteorological station 1 km from the study site, managed directly by the National Park administration at the La Cortijuela Botanical Garden. We considered January, February, and March as winter and July, August, and September as summer.

Foliar samples were collected in early March 2011 (winter) and mid‐July 2011 (summer). The PPM late‐instar larvae inflict the heaviest damage to pines in late winter (Battisti et al., 2015; Hódar et al., 2003), and the needles of the current year are completely flushed in July during the typical drought of the Mediterranean summer.

### 2.2 Experimental design and sampling of needles

Twenty‐four mature *iberica* and *nevadensis* trees, >45 years old and >6 m in height, were used as study cases (total *n* = 48). All sampled individuals were within a radius of approximately 500 m in the same valley at the same altitude. We randomly selected 12 trees of each subspecies attacked by PPMs (ATs), easily identified by their winter tents (2–4 per tree), and 12 trees of each subspecies with no signs of caterpillar attack (NATs). NATs and ATs were sampled simultaneously and within a short period of time (10:30–14:30) under sunny and constant light and temperature conditions to avoid large variations of metabolomes due to circadian rhythms (Kim, Choi, & Verpoorte, 2010; Rivas‐Ubach et al., 2013). A small branch exposed to the sun was removed from the NATs with a pruning pole. A small nonattacked branch (NAB) and a small attacked branch (AB), both exposed to the sun, were also removed from ATs (see Fig. S1). NABs were collected between 3 and 4 m away from the PPM attack ensuring at least 1 branch between the focus of attack and the sampled undamaged needles. These foliar samples will be referred as AT.NABs and AT.ABs, respectively. The youngest well‐developed needles from each sampled branch were quickly frozen in situ in liquid nitrogen for the metabolomic analyses.

Periodic outbreaks of PPMs occur in Spain and France with a return period of 5–9 years, and infestation is more unpredictable in some other areas of the Mediterranean Basin (Hódar, Zamora, & Cayuela, 2012; Li, Daudin, Piou, Robinet, & Jactel, 2015; Tamburini, Marini, Hellrigl, Salvadori, & Battisti, 2013). The intensity of the outbreaks, however, was variable and defoliation could be patchily distributed, even at sites heavily attacked by PPMs. A mild outbreak occurred in the study area in winter 2010, during which many trees were not affected by the PPM, and the PPM population declined after the outbreak during the winter of 2011, the year when the samples were collected.

Pine processionary moth attacks vary depending on pine species, and preferences can also vary from site to site (Jactel et al., 2015). Differences in attack preference between subspecies of pines, however, have not been documented. At our study site, *nevadensis* and *iberica* were equally attacked, and any possible differences would not affect our results because our selection of trees in the wild was based on the presence/absence of natural defoliation. This selection determines that our pines were not completely randomly assigned to the different folivory levels (NAT or AT). Moths in monospecific stands, as in our case, however, would mainly rely on visual cues to attack isolated or taller trees that were more likely to provide optimal microclimatic conditions (high solar radiation) for egg survival and successful larval development instead of on chemical differences between individuals (Jactel et al., 2015). The assignment of attacked/unattacked treatments by female moths when ovipositing can thus be reliably considered as a random selection of the prior chemistry of the trees. The NATs thus served as controls, and the AT.ABs and AT.NABs were used to determine the local and systemic responses to folivory, respectively, and to represent folivory levels (FLs).

### 2.3 Foliar processing for metabolomic analyses

Briefly, needles frozen in liquid nitrogen were lyophilized and stored in plastic cans at −20°C (Rivas‐Ubach et al., 2013). Samples were ground with a ball mill at 1,600 rpm for 8 min (Mikrodismembrator‐U; B. Braun Biotech International, Melsungen, Germany), producing a fine homogeneous powder that was stored at −80°C until the extraction of the metabolites (Rivas‐Ubach et al., 2013).

### 2.4 Extraction of metabolites for liquid chromatography‐mass spectrometry analyses

We followed a well‐established protocol for the extraction of polar and semi‐polar metabolites (t'Kindt, De Veylder, Storme, Deforce, & Van Bocxlaer, 2008) with minor modifications. First, two sets of 2‐ml centrifuge tubes were labeled: set A for the extractions and set B for the extracts from set A. One hundred milligram of the sample powder was weighed into each tube of set A, and 1 ml of MeOH/H_2_O (80:20) was added as an extractant. All tubes were vortexed for 15 min, sonicated for 5 min at room temperature and then centrifuged at 23,000 *g* for 5 min. After centrifugation, 0.6 ml of the supernatant from each tube of set A was transferred to the corresponding 2‐ml centrifuge tubes of set B. This procedure was performed twice for two extractions of each sample. The tubes of set B were then centrifuged at 23,000 *g* for 5 min, and the supernatants were collected by glass syringes, filtered through 0.22‐μm pore microfilters and transferred to a labeled set of HPLC vials. The vials were stored at −80°C until the LC‐MS analysis.

### 2.5 LC‐MS analyses

The metabolomic fingerprints of polar and semi‐polar metabolites of pine leaves were obtained by LC‐MS analyses. LC‐MS chromatograms were obtained using a Dionex Ultimate 3000 HPLC system (Thermo Fisher Scientific/Dionex RSLC, Dionex, Waltham, USA) coupled to an LTQ Orbitrap XL high‐resolution mass spectrometer (Thermo Fisher Scientific) equipped with an HESI II (heated electrospray ionization) source. A reversed‐phase C18 Hypersil gold column (150 × 2.1 mm, 3 μm particle size; Thermo Scientific) at 30°C was used for chromatography. The mobile phases consisted of acetonitrile (A) and water (0.1% acetic acid) (B). Both mobile phases were filtered and degassed for 10 min in an ultrasonic bath prior to use. The elution gradient began at 10% A (90% B) at a flow rate of 0.3 ml/min and was maintained for 5 min and then to 10% B (90% A) until minute 20 and held for 5 min. The initial proportions (10% A, 90% B) were gradually recovered over the next 5 min, and the column was washed and stabilized for 5 more minutes before injection of the next sample. The injection volume of the samples was 5 μl. All samples were injected twice, once with the HESI operating in negative ionization mode (−H) and once in positive ionization mode (+H). The Orbitrap mass spectrometer was operated in Fourier Transform Mass Spectrometry full‐scan mode with a mass range of 50–1,000 m/z and high‐mass resolution (60,000). The resolution and sensitivity of the spectrometer were monitored by injecting a standard of caffeine after every 10 samples, and the resolution was further monitored with lock masses. Blank samples were also analyzed during the sequence (see Rivas‐Ubach, Sardans, et al. (2016) for more details of the LC‐MS analyses).

### 2.6 Processing of LC‐MS chromatograms

The raw data files from the spectrometer were processed by MZmine 2.17 (Pluskal, Castillo, Villar‐Briones, & Orešič, 2010). Chromatograms from both positive and negative modes were separately baseline corrected, deconvoluted and aligned before the metabolic assignation. For each database generated (positive and negative), metabolites were identified by exact mass and retention time based on the measurements of the standards in the MS with total exact mass with the automatic assignation function of the software (see Table S1 for details and Table S2 for the identified metabolites). The numerical databases were then exported to a CSV sheet. Chromatogram builder and deconvolution algorithms may separate diverse ions with the same mass to ratio (m/z) into different variables due a slight shift in retention times, depending on the parameters established during chromatogram processing for obtaining the metabolomic data sets. All identified features corresponding to the same molecular compounds were thus summed to obtain only one variable per metabolite. Furthermore, most carbohydrates co‐eluted at very similar retention times with our chromatographic method in a reversed‐phase C18 column which makes impossible to differentiate them when they share the same exact mass. For this reason, some of the detected carbohydrates were thus classified into different groups based on their retention time and m/z (“hexoses” (Hex) for fructose, glucose, galactose and mannose; “pentoses” (Pent) for ribose, xylose and arabinose; “disaccharides” (Disacch) for saccharose and maltose; group 1 sugars (S1) for deoxy‐galactose, deoxy‐glucose and D‐fucose; group 2 sugars (S2) for raffinose and maltotriose; and group 3 sugars (S3) for arabitol and xylitol).

Metabolomic variables present in fewer than eight individuals of a cell factor were removed from the data set. Values of a specific variable threefold higher than the third quartile or threefold lower than the first quartile of each cell factor were considered as outliers and were subsequently treated as missing data.

The numerical values of the features extracted from the LC‐MC chromatograms correspond to the absolute peak areas of the chromatograms detected by the spectrometer. The integrated peak areas from the deconvoluted peak chromatograms do not reflect the real concentration as unit weight of metabolite per unit weight of the sample, but it is proportional to the concentration of the corresponding variable and so is suitable for comparative analyses, as demonstrated in several metabolomic studies (Lee & Fiehn, 2013; Mari et al., 2013; Rivas‐Ubach et al., 2014; Rivas‐Ubach, Sardans, et al. (2016)). We thus use the term *concentration* when referring to the relative concentrations of the metabolites among the factors studied (season, subspecies, and FL).

### 2.7 Data analyses

The average minimum and maximum temperatures and accumulated rainfall for winter and summer in Navacerrada and Sierra Nevada were compared with a *t* test (Table S3) to determine if the environmental conditions differed significantly between localities in each of the seasons.

For the metabolomic data, Shapiro–Wilk and Levene's tests were performed on each variable to assess normality and homogeneity of variances. All known variables met the assumptions for the posterior analyses of variance (ANOVAs), and any unidentified metabolomic variable that did not meet the assumptions was removed from the data set (127 variables removed from the initial data set of 8,492 variables). The data set for this study was thus composed of three independent factors, season (winter and summer), subspecies of *P. sylvestris* (*iberica* and *nevadensis*) and FL (NATs, AT.NABs, and AT.ABs), and contained 8,365 metabolomic variables, 72 of which were identified by our metabolite library (Table S2).

The metabolomic fingerprints for the *P. sylvestris* needles were subjected to a permutational multivariate analysis of variance (PERMANOVA) using Euclidean distances to test for differences in the overall metabolomes between seasons, subspecies, and FLs. The number of permutations was set at 10,000.

Univariant analyses consisted in one‐way ANOVAs, and Tukey's HSD post hoc tests were performed for each individual identified metabolite for each subspecies and season separately with FL as categorical factor (Table S4). The entire set of *p* values from the univariate analyses was subsequently submitted to a Benjamini–Hochberg correction test to control for false positives. The result of these one‐way ANOVAs showed whether there were statistically significant differences between the FLs (NATs vs. AT.NABs vs. AT.ABs) (Table S4).

The foliar metabolomic fingerprints of both pine subspecies were also subjected to principal component analysis (PCA) for each season separately. PCAs reduce the dimensionality of a data set into typically two dimensions (PC1 vs. PC2), and samples and variables are projected on the factor plane constrained by the two dimensions. We used PCA to understand the metabolomic trends of FLs and subspecies and to shed light in the relations between variables and study subjects. The score coordinates of the variables of the two first PCA axes were subjected to one‐way ANOVAs and Tukey's HSD post hoc tests to identify statistical differences among the metabolome fingerprinting of the different groups (subspecies and FLs) across the variability explained by the two first axes of the PCA (Rivas‐Ubach et al., 2013).

Additionally, we used Euclidean distances as a proxy to determine the distance between the metabolomes (metabolomic distances) among different groups of trees. The Euclidean distances between the metabolomic fingerprints of *iberica* and *nevadensis* were calculated for each individual tree within the same FL (each *iberica*‐NAT vs. each *nevadensis*‐NAT, each *iberica*‐AT.NAB vs. each *nevadensis‐*AT.NAB and each *iberica*‐AT.AB vs. each *nevadensis*‐AT.AB) (Fig. S2a–c). Those Euclidean distances are referred as “FL distances between subspecies” along the text. All the 144 FL distances between subspecies (12 *iberica* trees × 12 *nevadensis* trees for NATs, AT.NABs and AT.ABs) were subsequently submitted to a one‐way ANOVA considering winter and summer together and for each season separately (6 groups: 2 seasons × 3 FLs). ANOVAs were applied to identify for statistical differences between FL distances between subspecies. Euclidean distances between FLs within each subspecies (each NAT vs. each AT.NAB and each NAT vs. each AT.AB, separately for *iberica* and for *nevadensis*) were also calculated for each season (Fig. S2d,e). These second calculated Euclidean distances are referred as “FL distances within subspecies” along the text and were also submitted to a one‐way ANOVA considering winter and summer together (8 groups: 2 seasons × 2 distances × 2 subspecies) to identify differences between FL distances within subspecies.

Multivariate analyses such as PERMANOVAs, PCAs, and Euclidean‐distance calculations were performed using the complete data set (identified and nonidentified variables). The PERMANOVAs, one‐way ANOVAs, *t* tests, Tukey's post hoc tests, Shapiro‐Wilk tests, Levene's tests, PCAs, and Euclidean‐distance calculations were performed with R (R Core Team, 2013). The Shapiro‐Wilk tests, one‐way ANOVAs, *t* tests, Benjamini‐Hochberg correction, and Euclidean‐distance calculations were performed with the functions *shapiro.test*,* aov*,* p.adjust, t.test*, and *dist*, respectively, in the “stats” package (R Core Team, 2013). Tukey's HSD post hoc tests were performed by the *HSD.test* function of the “agricolae” package (de Mendiburu, 2015). Levene's tests were performed with the *leveneTest* function in the “car” package (Fox & Weisberg, 2011). The PERMANOVA was conducted with the *adonis* function in the package “vegan” (Oksanen et al., 2013). Using the regularized iterative PCA algorithm (Josse & Husson, 2013) with the *imputePCA* function of the “missMDA” package (Husson & Josse, 2015), the missing values of the data set were imputed before the PCA. Once the missing values were imputed, PCAs were performed with the *PCA* function of the “FactoMineR” package (Husson, Josse, Le, & Mazet, 2016).

## 3 RESULTS

Our univariate analyses contrasting the environmental data (temperature and accumulated rainfall) between Sierra Nevada and Navacerrada showed more contrasted environmental conditions between localities in summer compared to winter (Figure 3; Table S3). Average maximum temperatures were higher in both winter and summer in Sierra Nevada, the native locality for *nevadensis*, than Navacerrada, the native localities for *iberica*. Average minimum temperature and accumulated rainfall did not differ significantly between the two areas in winter, but the average minimum temperature was higher and accumulated rainfall was lower in Sierra Nevada than Navacerrada in summer.

The overall composition of the pine metabolomes changed significantly between seasons, subspecies and FLs (Table 1). PERMANOVA also found significant differences in all the factor interactions (season × subspecies; season × FL; subspecies × FL; season × subspecies × FL) (Table 1) indicating that the impact of any of the studied factor (season, subspecies, and FL) on the structure of the overall metabolomes of pines also depends on the level of the other two factors together and separately.

**Table 1:** Full factorial PERMANOVA model of the complete metabolomic data set considering all factors and interactions: season, subspecies, folivory level (FL), season × subspecies, season × FL, subspecies × FL, season × subspecies × FL, and residuals

	Degrees of freedom	Sums of squares	Mean squares	Pseudo‐*F*	*p*
Season	1	3.60 × 10^20^	3.60 × 10^20^	94.75	.0001
Subspecies	1	1.37 × 10^20^	1.37 × 10^20^	35.904	.0001
Folivory level (FL)	2	4.39 × 10^19^	2.20 × 10^19^	5.776	.0001
Season × Subspecies	1	8.55 × 10^19^	8.55 × 10^19^	22.469	.0001
Season × FL	2	4.74 × 10^19^	2.37 × 10^19^	6.231	.0001
Subspecies × FL	2	3.09 × 10^19^	1.55 × 10^19^	4.067	.0004
Season × Subspecies × FL	2	5.52 × 10^19^	2.76 × 10^19^	7.259	.0001
Residuals	132	5.02 × 10^20^	3.80 × 10^18^	0.39784	
Total	143	1.26 × 10^21^	1		

**Figure 4:**
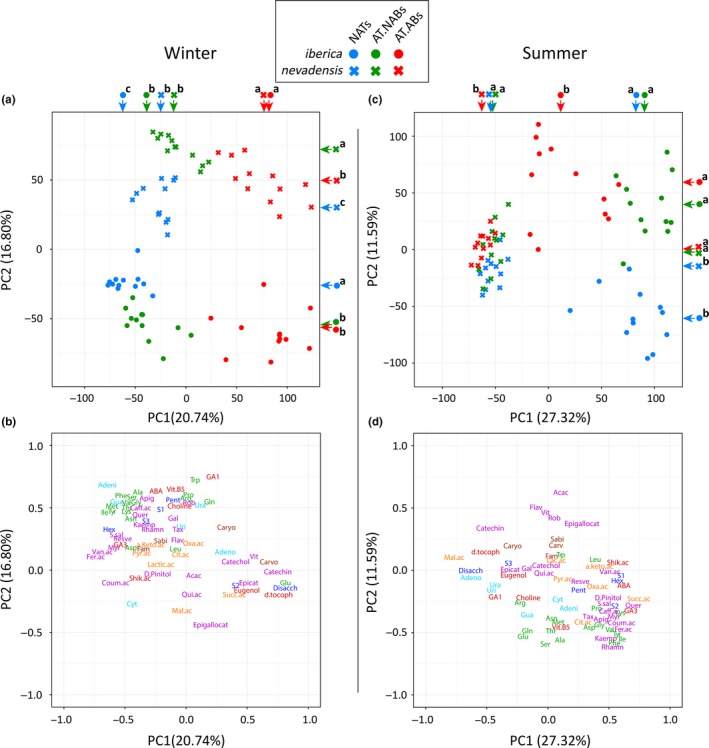
PC1 versus PC2 of the PCAs of the foliar metabolomes of *Pinus sylvestris* ssp. *iberica* and *P. sylvestris* ssp. *nevadensis* for winter and summer: case (a) and variable (b) plots of the PCA for winter, and case (c) and variable (d) plots of the PCA for summer. The folivory levels (FLs) are represented by different colors: blue, NATs; green, AT.NABs and red, AT.ABs. Crosses represent *nevadensis* and dots represent *iberica*. The colored arrows indicate the coordinate averages of PC1 and PC2 of each folivory level (FL) for *iberica* and *nevadensis*. Different letters beside the arrows indicate significant differences between FLs for each subspecies detected by Tukey's HSD post hoc tests (*p *< .05). Different metabolomic families are indicated by different colors: blue, sugars; green, amino acids; cyan, nucleotides; orange, organic acids associated with the tricarboxylic acid cycle (TCA); violet, phenolics; brown, terpenes; dark red, other secondary metabolites. Unidentified metabolites are not represented in the variable plot. Most metabolites are referenced by abbreviations: disaccharides (Disacch), hexoses (Hex), pentoses (Pent), group 1 sugars representing deoxy‐glucose, deoxy‐galactose, and D‐fucose (S1), group 2 sugars representing raffinose and maltotriose (S2), group 3 sugars representing xylitol and arabitol (S3), alanine (Ala), arginine (Arg), asparagine (Asn), aspartic acid (Asp), glutamic acid (Glu), glutamine (Gln), glycine (Gly), isoleucine (Ile), leucine (Leu), lysine (Lys), methionine (Met), phenylalanine (Phe), proline (Pro), serine (Ser), threonine (Thr), tryptophan (Trp), tyrosine (Tyr), valine (Val), adenine (Adeni), adenosine (Adeno), cytosine (Cyt), guanine (Gua), uridine (Uri), uracil (Ura), α‐ketoglutaric acid (a.keto.ac), citric acid (Cit.ac), lactic acid (Lac.ac), malic acid (Mal.ac), oxaloacetic acid (Oxa.ac), pyruvic acid (Pyr.ac), succinic acid (Succ.ac), acacetin (Acac), apigenin (Apig), caffeic acid (Caff.ac), catechin, catechol, coumaric acid (Coum.ac), D‐pinitol, epicatechin (Epicat), epigallocatechin (Epigallocat), ferulic acid (Fer.ac), galangin (Gal), kaempferol (Kaemp), myricetin (Myr), quercetin (Quer), quinic acid (Qui.ac), resveratrol (Resve), rhamnetin (Rhamn), robinetin (Rob), sodium salicylate (S.sal), taxifolin (Tax), vitexin (Vit), vanillic acid (Van.ac), 5,7‐dihydroxy‐3,4,5‐trimethoxyflavone (Flavone: Flav), choline, δ‐tocopherol (d.tocoph), eugenol, vitamin B5 (Vit.B5), shikimic acid (Shik.ac), sabinene (Sabi), carvone (Carvo), caryophyllene (Caryo), farnesol (Farn), abscisic acid (ABA), gibberellic acid 1 (GA1), and gibberellic acid 3 (GA3)

The PCA performed in winter, when the PPM is active, showed that the different levels of FLs of both subspecies followed the same trend along the PC1 suggesting thus certain grade of similarity on the metabolomic responses to PPM attack in both subspecies (Figure 4a). PC2 of the PCA in winter displayed clear separation between subspecies indicating their different overall metabolome composition (Figure 4a). PCA summer showed different trends for subspecies and FLs. Subspecies still clustered separately but along PC1 (Figure 4c). Although not as clear as in the winter PCA, FLs of both subspecies also followed similar trends along PC1 and PC2 (Figure 4c). For both subspecies, NATs and AT.NABs were separated from the AT.ABs along PC1 while PC2 separated the NATs from the AT.NABs and AT.ABs (Figure 4c). Additionally, FLs of *iberica* in summer were clearly more segregated among them than FLs of *nevadensis* (Figure 4c). These results indicate that the metabolome shifts between FLs were larger in *iberica* than *nevadensis* in summer.

Regarding the relations between the metabolomic variables and study subjects, the PCA showed that the concentrations of most amino acids, sugars, phenolic compounds, and terpenes tended to be higher in *nevadensis* than *iberica* needles in winter (Figure 4a,b). The AT.ABs of both subspecies had the highest concentrations of vitexin, catechin, carvone, disaccharides, and δ‐tocopherol, and the NATs of both subspecies had the highest concentrations of amino acids, nitrogenous bases (adenine, guanine, and cytosine), and most phenolic compounds (Figure 4a,b; Table S4). Nevertheless, the concentrations of amino acids, phenolic compounds, and most sugars were highest in *iberica* in summer, especially for the NATs and AT.NABs (Figure 4c,d). The NATs of both subspecies had higher concentrations of most amino acids, organic acids, sugars, nitrogenous bases, and most phenolics relative to the ATs (Figure 4c,d; Table S4).

**Figure 5:**
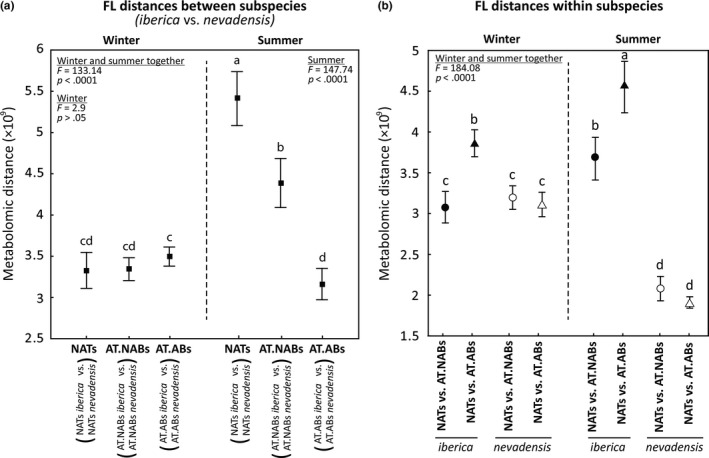
Metabolomic distance (Euclidean distances) between *iberica* and *nevadensis* metabolomes for each folivory level (FL; NATs, AT.NABs, AT.ABs) and season (winter, summer) (a). Metabolomic distances between NATs versus AT.NABs (circles) and NATs versus AT.ABs (triangles) within each subspecies and for each season (b). Solid and open circles and triangles represent, respectively, to *iberica* and *nevadensis* in panel b. Values represent the distance means ± 3 *SE*. Different letters denote significant differences identified by one‐way ANOVAs and HSD post hoc tests (*p* < .05). The ANOVA for panel b used both winter and summer data

In winter, the FL distances between subspecies did not differ significantly (Figure 5a). In summer, however, the distance for *iberica* NATs versus *nevadensis* NATs was highest, whereas the distance for *iberica*‐AT.ABs versus *nevadensis*‐AT.ABs was lowest (Figure 5a). Those results indicate that, in summer, when the environmental conditions between Sierra Nevada and Navacerrada are more contrasted (Figure 3), NATs between subspecies had more contrasted metabolomes compared to AT.ABs which presented the smallest metabolic differences between *iberica* and *nevadensis*. In both winter and summer, FLs distances within *nevadensis* did not differ significantly among them (Figure 5b). Differently, *iberica* had higher NATs versus AT.ABs distance than NATs versus AT.NABs distance in both seasons (Figure 5b). Additionally, both NATs versus AT.ABs and NATs versus AT.NABs distances for *iberica* were significantly higher in summer compared to winter (Figure 5b). Nonetheless, FL distances within *nevadensis* were higher in winter, when the PPM is present, than in summer (Figure 5b). Those results indicated larger metabolomic difference between subspecies to PPM attack in summer than in winter.

## 4 DISCUSSION

The conclusions of the present study directly rely on the comparison of the metabolic responses of two coexisting Scot pine subspecies in Sierra Nevada, one native (*nevadensis*) and one introduced (*iberica*). Although this study does not include *iberica* samples from its native region (Navacerrada) to definitely corroborate our conclusions, the multivariate analyses of the metabolomes of both subspecies clearly indicate closer metabolomic responses to PPM attack between subspecies and more distant metabolomic responses to the marked seasonality of Sierra Nevada.

### 4.1 Close metabolic responses to folivory attack

Defoliation by PPM occurs during winter (Battisti et al., 2015); interestingly although different FLs presented different overall metabolome structure in both subspecies (Table 1), the winter metabolomes of the subspecies tended to converge in response to local caterpillar attack. PC1 of the winter PCA case plot (Figure 4b) separated the FLs, and AT.ABs and AT.NABs of both subspecies clearly had the same direction respect to the NATs along PC1 suggesting that both subspecies presented certain similarity in the metabolomic responses to folivory. In particular, the AT.ABs of both subspecies had the highest concentrations of vitexin, catechin, carvone, disaccharides, and δ‐tocopherol (Figure 4a,b; Table S4), metabolites which have been directly associated with folivory. Folivory causes oxidative stress in plants (Bi & Felton, 1995; Ruuhola & Yang, 2006), and δ‐tocopherol and flavonoids such as vitexin and catechin have been commonly considered as important antioxidants in plants (Apel & Hirt, 2004; Khorasani Esmaeili, Mat Taha, Mohajer, & Banisalam, 2015; Kim et al., 2005; Raman et al., 2016; Rice‐Evans, Miller, & Paganga, 1996; Singh, Sahu, & Sharma, 2017). Hernández, Alegre, Van Breusegem, and Munné‐Bosch (2009), however, reviewed the role of flavonoids in plants and concluded that the antioxidant function of flavonoids in plants is still a matter of debate due the lack of a strong spatiotemporal correlation between oxidative stress and flavonoid oxidation, even though different species of flavonoids can accumulate in in vitro plants under oxidative stress in response to diverse biotic and abiotic stressors. On the other hand, transgenic plants that over‐express the genes that code for flavonoids have recently been used to produce progeny with improved antifungal and antioxidative properties (Mierziak et al., 2014; Ravensdale et al., 2014). Although most studies agree that flavonoids have strong antioxidant properties in plants, further research is necessary to deeply decipher the spatiotemporal mechanism of flavonoids with plant oxidation stress. Carvone has been described as a terpene with repellent and antifeedant properties in pines (Schlyter, Smitt, Sjödin, Högberg, & Löfqvist, 2004). Some studies have demonstrated higher rates of sucrose secretion in damaged leaves that can attract more insect visitors (Ness, 2003; Rivas‐Ubach et al., 2014) and that can account for the higher concentrations of disaccharides in the AT.ABs (Figure 4a,b; Table S4). A more detailed discussion of the functional roles of the metabolomic differences between FLs in both subspecies has been published elsewhere (Rivas‐Ubach, Sardans, et al., 2016). Additionally, in summer, when the temperatures and drought in Sierra Nevada are more extreme than in Navacerrada (Figures 2 and 3), the AT.ABs distance between subspecies have similar values respect to winter but NATs distance between subspecies increased significantly (Figure 5a). These results also suggest that the AT.ABs of both subspecies had more common metabolomic responses in summer, likely for coping with the injuries from the last folivory (Rivas‐Ubach, Sardans, et al., 2016).

The convergence of the foliar metabolomes of the subspecies in the local responses to PPM attack (AT.ABs) is consistent with the notion that several metabolic responses induced by herbivorous attack may be evolutionarily conserved (Carrillo‐Gavilán et al., 2015). Overall metabolomic responses to PPM, however, have been reported to differ in three pine species, but the responses were directly related to the phylogeny of the pines, suggesting again particular evolutionarily conserved responses to PPM attack (Rivas‐Ubach, Hódar, et al., 2016). Our study analyzed two subspecies of *P. sylvestris* that are historically more closely related than individuals belonging to different species, so the convergence between the subspecies in metabolomic responses to herbivorous attack in our analyses of the metabolomic fingerprints also suggests a strong genetic component determining the responses. If metabolomes track phylogeny, we would expect that distantly related species would produce divergent metabolomic responses to herbivorous attack. Future research analyzing different plant species attacked by the same folivore would be necessary to strongly support this statement.

### 4.2 Distant metabolomic responses to summer drought

Despite belonging to the same species, the seasonal overall metabolomic differences between *iberica* and *nevadensis* (Table 1) may have been due to both the genetic component between subspecies and to the atypically extreme environment experienced by the introduced *iberica* in summer (Figures 2 and 3; Table S3). We found diverse metabolomic evidence that *iberica* in Sierra Nevada (introduced) experienced a more extreme environment in summer relative to *nevadensis* (native). First, FLs distances between subspecies did not differ significantly among them in winter, but in summer, when the PPM is not present, NATsc distance between subspecies was significantly higher than all FLs distances between subspecies in winter (Figure 5a). This result suggests that AT.ABs of both subspecies retain some common responses in summer to PPM attack, but NATs, which do not need to cope with folivory injuries, are metabolically more different between subspecies. This trend was not found in winter when the climatic conditions are more similar between Sierra Nevada and Navacerrada, but maximum temperatures are higher and rainfall is lower in Sierra Nevada during summer, so drought is more severe in Sierra Nevada than Navacerrada (Figure 3; Table S3). Second, the PCA for summer indicated that metabolomic variation between the FLs was lower for *nevadensis* than for *iberica*. The metabolomes of the *iberica* NATs, AT.NABs and AT.ABs were more clearly separated than the FLs of the *nevadensis* trees in the multidimensional space of the PCA (Figure 4a). This PCA thus indicated that the *iberica* responses to PPM attack were larger than *nevadensis* in summer, when the PPM is not present. Third and also supported by the PCA, the FLs distances within subspecies in summer presented larger differences between *iberica* and *nevadensis* than in winter, when only the *iberica* NATs versus AT.ABs distance was significantly larger (Figure 5b). This divergence between *nevadensis* and *iberica* in summer clearly supports the idea that the environment was more influential in summer in *iberica*, the subspecies introduced to Sierra Nevada. Fourth, independently of the FL, the concentrations of sugars, phenolics, and amino acids such as proline (Figure 4c,d) in summer were higher in *iberica* than *nevadensis* needles, also suggesting that *iberica* experienced more stress in summer. In previous ecometabolomic studies, we have also observed increases in sugar, amino acid, and phenolic concentrations in different plant species during the dry summers of the Mediterranean climate (Rivas‐Ubach et al., 2012, 2014; Rivas‐Ubach, Barbeta, et al., 2016). Proline is an important osmoprotectant in plants (Szabados & Savouré, 2010), so the higher proline concentrations in the needles of *iberica* compared to *nevadensis* also suggest that this subspecies may be facing more intense drought conditions in Sierra Nevada than those in its native range in Navacerrada (Figure 3). The higher concentrations of phenolic compounds and sugars in *iberica* than *nevadensis* needles have been also widely reported as protective mechanisms against water deficit (Hura, Hura, & Grzesiak, 2008; Ingram & Bartels, 1996; Rivas‐Ubach et al., 2014).

Our results thus indicated that *nevadensis* experienced less metabolomic variation in summer, when warm temperatures and drought are more prominent, than *iberica*. Our metabolomic results suggest thus that the native populations of Scots pine are better adapted than the introduced populations to the environmental conditions in Sierra Nevada (Herrero & Zamora, 2014), anticipating a more stressful abiotic environment for *iberica* populations as climatic belts progress poleward. The PPM is present in the *iberica* populations, but the intensity and frequency of defoliaton has been increasing altitudinally and latitudinally during recent decades (Battisti et al., 2005; Hódar & Zamora, 2004). The maximum temperatures are about 4°C higher, and the accumulated rainfall is 40% lower in Sierra Nevada than Navacerrada, so the conditions are significantly warmer and drier than in the natural range of *iberica* (Figure 3; Table S3). The increases in the synthesis of some metabolites necessary to maintain ecophysiological function under drought environmental conditions (Gaspar et al., 2002) indicate that the more extreme summer environmental conditions in Sierra Nevada compared to Navacerrada contributed substantially to the large shifts in the foliar metabolome of *iberica* pines. Additionally, planted (*iberica*) populations of *P. sylvestris* have declined significantly in southeastern areas of Sierra Nevada where summers are even more extreme than in our study site (Cerrillo, Varo, Lanjeri, & Clemente, 2007; Guada, Camarero, Sánchez‐Salguero, & Cerrillo, 2016), supporting our results. Our metabolomic data thus support our hypothesis that the planted populations of *iberica* in Sierra Nevada are outside their natural environmental niche, even though *iberica* and *nevadensis* belong to the same species, and therefore need to cope with the more extreme summer conditions, forcing trees to produce larger shifts in their metabolomes (Shao et al., 2007). We thus predict that *iberica* populations will have an uncertain future as warmer and drier conditions in combination with severer defoliation continue to progress poleward.

## 5 CONCLUSIONS

The metabolomes of the two closely related subspecies of Scots pine tended to have similar local responses to herbivorous attack, suggesting that some metabolic pathways associated with folivory may have been evolutionarily conserved.

The environmental conditions of summer are significantly more extreme in Sierra Nevada than in Navacerrada, with significant higher temperatures and more severe droughts in summer. Both pine subspecies analyzed in this study coexist in the same environment in Sierra Nevada, but the metabolomic differences between them were more pronounced in summer and the concentrations of metabolites typically associated with drought stress were higher in *iberica* (introduced subspecies) than *nevadensis* (native subspecies).

The metabolomic results of both pine subspecies suggest that the divergence between the summer *iberica* and *nevadensis* metabolomes relative to winter is, in part, associated with the natural distributions of the subspecies.

A longer period of local adaptation likely provided *nevadensis* with a metabolism that is better adapted to drought conditions than is the metabolism of *iberica*, which is subjected to more drought and higher temperatures in Sierra Nevada than it experiences in its natural habitat. These more extreme conditions for *iberica* may account for the larger shifts in their metabolomes to maintain physiological homeostasis. We anticipate an uncertain future for *iberica* populations in Sierra Nevada with the warmer and drier conditions expected during the forthcoming decades.

Eco‐metabolomic techniques are potential tools to understand long‐time ecological processes rather than only biochemical processes.

## CONFLICT OF INTEREST

None declared.

## AUTHOR CONTRIBUTIONS

AR‐U wrote the manuscript. AR‐U, JS, JAH, AG, and JP designed and performed the research, collected the samples, and interpreted the data. AR‐U, JS, JAH, and JG‐P analyzed the data. MO and OU obtained the metabolomic fingerprints of the samples. AR‐U, JS, JAH, JG‐P, AG, LP‐T, MO, OU, and JP read and approved the final version of the manuscript as well as contributed addressing properly all concerns from reviewers.
