# Supplementary material for: Close and distant: Contrasting the metabolism of two closely related subspecies of Scots pine under the effects of folivory and summer drought
Source: Ecol Evol. 2017 Sep 25;7(21):8976–88. doi: 10.1002/ece3.3343 (PMC5677489; doi:10.1002/ece3.3343)

**Supplementary Information**

**Table S1.** LC-MS chromatogram processing. Chromatograms were obtained by liquid chromatography (LC-MS) and processed by MZmine 2.17. The following table summarizes the different processes and parameters applied to the Scots pine chromatograms.

|  |  | | (+H) Chromatograms |  | (-H) Chromatograms |
| --- | --- | --- | --- | --- | --- |
| **1** | **Baseline correction** | |  |  |  |
|  | Chromatogram type | | TIC |  | TIC |
|  | MS level | | 1 |  | 1 |
|  | Smoothing | | 10E6 |  | 10E6 |
|  | Asymmetry | | 0.001 |  | 0.001 |
| **2** | **Mass detection** (Exact Mass) | |  |  |  |
|  | Noise level | | 3.0 × 10^5^ |  | 1.0 × 10^5^ |
| **3** | **Chromatogram builder** | |  |  |  |
|  | Minimum time span | | 0.05 |  | 0.05 |
|  | Minimum height | | 25 000 |  | 25 000 |
|  | m/z tolerance | | 0.0005 |  | 0.0005 |
| **4** | **Smoothing** | |  |  |  |
|  | Filter width | | 5 |  | 5 |
| **5** | **Chromatogram deconvolution (Local) minimum search)** | |  |  |  |
|  | Chromatographic threshold | | 65% |  | 65% |
|  | Search minimum in RT range (min) | | 0.05 |  | 0.1 |
|  | Minimum relative height | | 5.0% |  | 5.0% |
|  | Minimum absolute height | | 30 000 |  | 30 000 |
|  | Min ratio of peak top/edge | | 2 |  | 2 |
|  | Peak duration range | | 0.0-2.0 |  | 0.0-2.0 |
| **6** | **Chromatogram alignment (Alignment)** | |  |  |  |
|  | m/z tolerance | | 0.0005 |  | 0.0005 |
|  | Weight for m/z | | 80 |  | 80 |
|  | RT tolerance | | 0.2 |  | 0.2 |
|  | Weight for RT | | 20 |  | 20 |
| **7** | **Gap filling (Peak Finder)** | |  |  |  |
|  | Intensity tolerance | | 20% |  | 20% |
|  | m/z tolerance | | 0.0005 |  | 0.0005 |
|  | Retention time tolerance | | 0.1 |  | 0.1 |
|  | RT correction | | marked |  | marked |
| **8** | **Metabolite Assignation** | |  |  |  |
|  | m/z tolerance | | 0.0005 |  | 0.0005 |
|  | RT tolerance (depending on the time on the chromatogram) | Up to 10 min | 0.25 |  | 0.25 |
|  |  | 10-30 min | 0.5 |  | 0.5 |
| **9** | **Filtering** | |  |  |  |
|  | Minimum peaks in a row | | 8 |  | 8 |

**Table S2.** Mass-to-charge ratio (m/z) and retention time (RT) of the deconvoluted ions assigned to metabolites with MZmine v.2.17. The assignment of the metabolites was based on the exact mass and RT of the standards for both negative and positive ionization modes. RT, m/z and peak range of the standards are shown in the table. Standards with double peaks in the chromatograms and ions representing fragments of the molecular compound are marked. The errors for m/z and RT of the ions assigned to metabolites relative to the errors for m/z and RT of the standards are shown.

| Ionization mode |  | m/z and RT of each  ion assigned  in MZmine v.2.12 | |  | m/z, RT, peak range of ions from standards measured in the LC-MS Orbitrap | | | | |  | Error of m/z and RT (MZmine deconvoluted ions vs. standard ions) | |
| --- | --- | --- | --- | --- | --- | --- | --- | --- | --- | --- | --- | --- |
|  | Metabolite | m/z | RT (min) |  | m/z | RT (min) | Peak range (min) | Standard with double peak | Fragment |  | m/z error | RT error (min) |
| Negative | 5_7_dihydroxy_3_4_5_trimethoxyflavone (Flavone) | 343.0823 | 16.61 |  | 343.08252 | 16.42 | 16.3 - 16.6 |  |  |  | -0.00022 | 0.19 |
|  | 5_7_dihydroxy_3_4_5_trimethoxyflavone (Flavone) | 343.0829 | 16.7 |  | 343.08252 | 16.42 | 16.3 - 16.6 |  |  |  | 0.00038 | 0.28 |
|  | Alpha-ketoglutaric acid | 145.0149 | 1.53 |  | 145.01483 | 1.65 | 1.57 - 1.87 |  |  |  | 0.00007 | -0.12 |
|  | Alpha-ketoglutaric acid | 145.0149 | 1.69 |  | 145.01483 | 1.65 | 1.57 - 1.87 |  |  |  | 0.00007 | 0.04 |
|  | Abscisic acid (ABA) | 263.1294 | 13.53 |  | 263.12888 | 13.56 | 13.4 - 13.8 |  |  |  | 0.00052 | -0.03 |
|  | Acacetin | 283.0616 | 16.8 |  | 283.06134 | 16.87 | 16.74 - 17.2 |  |  |  | 0.00026 | -0.07 |
|  | Apigenin | 269.0461 | 14.61 |  | 269.04623 | 14.63 | 14.145 - 15 |  |  |  | -0.00013 | -0.02 |
|  | Arginine | 173.105 | 1.33 |  | 173.10448 | 1.23 | 1.13 - 1.46 |  |  |  | 0.00052 | 0.1 |
|  | Aspartic acid | 132.0308 | 1.4 |  | 132.03027 | 1.41 | 1.31 - 1.75 |  |  |  | 0.00053 | -0.01 |
|  | Catechin | 289.0723 | 3.3 |  | 289.07254 | 3.39 | 2.71 - 3.91 |  |  |  | -0.00024 | -0.09 |
|  | Catechin | 289.0723 | 3.42 |  | 289.07254 | 3.39 | 2.71 - 3.91 |  |  |  | -0.00024 | 0.03 |
|  | Catechin | 289.0722 | 3.17 |  | 289.07254 | 3.39 | 2.71 - 3.91 |  |  |  | -0.00034 | -0.22 |
|  | Chlorogenic acid | 353.0885 | 2.99 |  | 353.08792 | 3.02 | 2.93 - 3.61 |  |  |  | 0.00058 | -0.03 |
|  | Citric acid | 173.0098 | 1.74 |  | 173.00925 | 1.77 | 1.72 - 2.01 |  | yes |  | 0.00055 | -0.03 |
|  | Citric acid | 191.0203 | 1.74 |  | 191.01976 | 1.77 | 1.72 - 2.01 |  |  |  | 0.00054 | -0.03 |
|  | Coumaric acid | 163.0406 | 10.94 |  | 163.04065 | 11.3 | 11.17 - 11.45 |  |  |  | -0.00005 | -0.36 |
|  | Coumaric acid | 163.0406 | 10.94 |  | 163.04065 | 11.3 | 11.17 - 11.45 |  |  |  | -0.00005 | -0.36 |
|  | Deoxy-glucose - Deoxy-galactose - D-Fucose (group 1 sugars) | 163.0619 | 1.68 |  | 163.06145 | 1.45 | 1.40 - 1.53 |  |  |  | 0.00045 | 0.23 |
|  | Deoxy-glucose - Deoxy-galactose - D-Fucose (group 1 sugars) | 163.0619 | 1.45 |  | 163.06145 | 1.45 | 1.40 - 1.53 |  |  |  | 0.00045 | 0.00 |
|  | Disaccharides | 341.1074 | 1.43 |  | 341.10776 | 1.42 | 1.33 - 1.74 |  |  |  | -0.00036 | 0.01 |
|  | D-Pinitol | 193.0721 | 1.41 |  | 193.07152 | 1.38 | 1.22 - 2.94 |  |  |  | 0.00058 | 0.03 |
|  | D-Pinitol | 193.072 | 1.53 |  | 193.07152 | 1.38 | 1.22 - 2.94 |  |  |  | 0.00048 | 0.15 |
|  | d-tocopherol | 401.1293 | 1.52 |  | 401.12906 | 1.35 | 1.21 - 1.45 |  |  |  | 0.00028 | 0.17 |
|  | Epicatechin | 289.0723 | 5.15 |  | 289.07254 | 5.33 | 4.05 - 6.14 | yes |  |  | -0.00024 | -0.18 |
|  | Epigallocatechin | 305.0674 | 2.56 |  | 305.06772 | 2.64 | 1.43 - 3.02 | yes |  |  | -0.00032 | -0.08 |
|  | Epigallocatechin | 305.0673 | 2.53 |  | 305.06772 | 2.64 | 1.43 - 3.02 | yes |  |  | -0.00042 | -0.11 |
|  | Ferulic acid | 193.0511 | 10.41 |  | 193.05095 | 10.59 | 10.35 - 10.81 |  |  |  | 0.00015 | -0.18 |
|  | Ferulic acid | 193.0512 | 10.54 |  | 193.05095 | 10.59 | 10.35 - 10.81 |  |  |  | 0.00025 | -0.05 |
|  | Gibberellic acid (GA3) | 345.1336 | 10.37 |  | 345.13391 | 10.63 | 10.47 - 10.76 |  |  |  | -0.00034 | -0.26 |
|  | Gibberellic acid (GA3) | 345.1335 | 11.02 |  | 345.13391 | 10.63 | 10.47 - 10.76 |  |  |  | -0.00038 | 0.39 |
|  | Gibberellic acid (GA3) | 345.1336 | 10.88 |  | 345.13391 | 10.63 | 10.47 - 10.76 |  |  |  | -0.00036 | 0.25 |
|  | Glutamic acid | 146.0466 | 1.4 |  | 146.04614 | 1.42 | 1.36 - 1.81 |  |  |  | 0.00046 | -0.02 |
|  | Glutamic acid | 146.0465 | 1.53 |  | 146.04614 | 1.42 | 1.36 - 1.81 |  |  |  | 0.00036 | 0.11 |
|  | Glutamine | 145.0625 | 1.38 |  | 145.062 | 1.35 | 1.28 - 1.5 |  |  |  | 0.00050 | 0.03 |
|  | Hexoses | 143.0351 | 1.38 |  | 143.03496 | 1.43 | 1.4 - 1.6 |  | yes |  | 0.00015 | -0.05 |
|  | Hexoses | 179.0561 | 1.37 |  | 179.05595 | 1.43 | 1.4 - 1.6 |  |  |  | 0.00017 | -0.06 |
|  | Hexoses | 143.0351 | 1.55 |  | 143.03496 | 1.43 | 1.4 - 1.6 |  | yes |  | 0.00014 | 0.12 |
|  | Hexoses | 179.0561 | 1.54 |  | 179.05595 | 1.43 | 1.4 - 1.6 |  |  |  | 0.00015 | 0.11 |
|  | Isoleucine | 130.0877 | 1.74 |  | 130.0874 | 1.52 | 1.42 - 1.69 |  |  |  | 0.00034 | 0.22 |
|  | Kaempferol | 285.041 | 14.76 |  | 285.04059 | 14.81 | 14.65 - 15.00 |  |  |  | 0.00041 | -0.05 |
|  | Kaempferol | 285.0411 | 15.22 |  | 285.04059 | 14.81 | 14.65 - 15.00 |  |  |  | 0.00051 | 0.41 |
|  | Lactic acid | 89.02495 | 1.52 |  | 89.02454 | 1.74 | 1.48 - 2.1 |  |  |  | 0.00041 | -0.22 |
|  | Lactic acid | 89.02493 | 1.78 |  | 89.02454 | 1.74 | 1.48 - 2.1 |  |  |  | 0.00039 | 0.04 |
|  | Leucine | 130.0876 | 1.57 |  | 130.08746 | 1.67 | 1.48 - 1.80 |  |  |  | 0.00014 | -0.1 |
|  | Leucine | 130.0876 | 1.71 |  | 130.08746 | 1.67 | 1.48 - 1.80 |  |  |  | 0.00014 | 0.04 |
|  | Malic acid | 115.0043 | 1.47 |  | 115.0047 | 1.51 | 1.44 - 1.74 | yes | yes |  | -0.00040 | -0.04 |
|  | Malic acid | 133.0152 | 1.46 |  | 133.0155 | 1.51 | 1.44 - 1.73 | yes |  |  | -0.00030 | -0.05 |
|  | Malic acid | 115.0043 | 1.64 |  | 115.0047 | 1.51 | 1.44 - 1.74 | yes | yes |  | -0.00040 | 0.13 |
|  | Malic acid | 133.0153 | 1.48 |  | 133.0155 | 1.51 | 1.44 - 1.73 | yes |  |  | -0.00020 | -0.03 |
|  | Malic acid | 133.0158 | 1.55 |  | 133.0155 | 1.51 | 1.44 - 1.73 | yes |  |  | 0.00030 | 0.04 |
|  | Myricetin | 317.0308 | 12.41 |  | 317.03091 | 12.47 | 12.33 - 13.2 |  |  |  | -0.00011 | -0.06 |
|  | Oxaloacetic acid | 133.0512 | 1.79 |  | 133.05095 | 1.96 | 1.87 - 2.1 |  |  |  | 0.00025 | -0.17 |
|  | Oxaloacetic acid | 133.0512 | 1.96 |  | 133.05095 | 1.96 | 1.87 - 2.1 |  |  |  | 0.00025 | 0 |
|  | Pantothenic acid (Vit B5) | 218.1034 | 1.97 |  | 218.10324 | 2.01 | 1.79 - 2.18 |  |  |  | 0.00018 | -0.04 |
|  | Pentoses | 149.0462 | 1.44 |  | 149.04564 | 1.43 | 1.34 - 1.71 |  |  |  | 0.00056 | 0.01 |
|  | Phenylalanine | 164.0721 | 1.91 |  | 164.07162 | 1.85 | 1.75 - 2.0 |  |  |  | 0.00050 | 0.06 |
|  | Protocatechuic acid | 153.02 | 2.57 |  | 153.01987 | 2.59 | 2.3 - 2.75 |  |  |  | 0.00013 | -0.02 |
|  | Protocatechuic acid | 153.0199 | 2.62 |  | 153.01987 | 2.59 | 2.3 - 2.75 |  |  |  | 0.00003 | 0.03 |
|  | Pyruvic acid | 87.0093 | 1.69 |  | 87.00888 | 1.65 | 1.49 - 1.81 |  |  |  | 0.00042 | 0.04 |
|  | Pyruvic acid | 87.0093 | 1.48 |  | 87.00888 | 1.65 | 1.49 - 1.81 |  |  |  | 0.00042 | -0.17 |
|  | Quercetin | 301.036 | 13.7 |  | 301.03598 | 13.73 | 13.6 - 14.01 |  |  |  | 0.00002 | -0.03 |
|  | Quercetin | 301.0358 | 13.66 |  | 301.03598 | 13.73 | 13.6 - 14.01 |  |  |  | -0.00018 | -0.07 |
|  | Quinic acid | 191.0556 | 1.42 |  | 191.05588 | 1.47 | 1.39 - 1.73 |  |  |  | -0.00028 | -0.05 |
|  | Quinic acid | 191.0554 | 1.59 |  | 191.05588 | 1.47 | 1.39 - 1.73 |  |  |  | -0.00048 | 0.12 |
|  | Raffinose pentahydrate – Maltotriose (group 2 sugars) | 503.1614 | 1.37 |  | 503.16119 | 1.37 | 1.34 - 1.47 |  |  |  | 0.00024 | 0.00 |
|  | Resveratrol | 227.0719 | 13.01 |  | 227.07216 | 13.09 | 12.92 - 13.21 |  |  |  | -0.00026 | -0.08 |
|  | Rhamnetin | 315.0516 | 15.9 |  | 315.05130 | 15.95 | 15.8 - 16.3 |  |  |  | 0.00030 | -0.05 |
|  | Shikimic acid | 173.0461 | 1.63 |  | 173.04553 | 1.63 | 1.31 - 1.87 |  |  |  | 0.00057 | 0.00 |
|  | Shikimic acid | 173.0461 | 1.58 |  | 173.04553 | 1.63 | 1.31 - 1.87 |  |  |  | 0.00057 | -0.05 |
|  | Salicylic acid | 137.0249 | 10.37 |  | 137.02457 | 10.51 | 10.36 -10.83 |  |  |  | 0.00033 | -0.14 |
|  | Succinic acid | 117.0199 | 1.55 |  | 117.01962 | 1.74 | 1.69 - 1.84 |  |  |  | 0.00028 | -0.19 |
|  | Succinic acid | 117.02 | 1.79 |  | 117.01962 | 1.74 | 1.69 - 1.84 |  |  |  | 0.00038 | 0.05 |
|  | Succinic acid | 99.0093 | 1.59 |  | 99.00911 | 1.74 | 1.69 - 1.84 |  | yes |  | 0.00019 | -0.15 |
|  | Taxifolin | 303.0516 | 10.96 |  | 303.05127 | 11.09 | 10.89 - 22.24 |  |  |  | 0.00033 | -0.13 |
|  | Tryptophan | 203.0831 | 2.42 |  | 203.08261 | 2.36 | 2.25 - 2.75 |  |  |  | 0.00049 | 0.06 |
|  | Tryptophan | 203.083 | 2.43 |  | 203.08261 | 2.36 | 2.25 - 2.75 |  |  |  | 0.00039 | 0.07 |
|  | Uracil | 111.0203 | 1.54 |  | 111.02009 | 1.49 | 1.43 - 1.85 |  |  |  | 0.00021 | 0.05 |
|  | Vanillic acid | 167.0356 | 4.5 |  | 167.03564 | 4.67 | 4.45 - 4.9 |  |  |  | -0.00004 | -0.17 |
|  | Vitexin | 431.0994 | 11.58 |  | 431.09955 | 11.5 | 10.95 - 11.57 |  |  |  | -0.00015 | 0.08 |
|  | Vitexin | 431.0994 | 11.67 |  | 431.09955 | 11.5 | 10.95 - 11.57 |  |  |  | -0.00015 | 0.17 |
|  | Xylitol - Arabitol  (group 3 sugars) | 151.0612 | 1.41 |  | 151.06154 | 1.4 | 1.31 - 1.67 |  |  |  | -0.00034 | 0.01 |
|  | Xylitol - Arabitol  (group 3 sugars) | 151.0612 | 1.58 |  | 151.06154 | 1.4 | 1.31 - 1.67 |  |  |  | -0.00034 | 0.18 |
| Positive | 5_7_dihydroxy_3_4_5_trimethoxyflavone (Flavone) | 345.0965 | 16.68 |  | 345.09595 | 16.39 | 16.3 - 16.5 |  |  |  | 0.00055 | 0.29 |
|  | 5_7_dihydroxy_3_4_5_trimethoxyflavone (Flavone) | 345.0954 | 16.43 |  | 345.09595 | 16.39 | 16.3 - 16.5 |  |  |  | -0.00055 | 0.04 |
|  | Abscisic acid (ABA) | 247.1323 | 13.59 |  | 247.13243 | 13.57 | 13.45 - 13.76 |  | yes |  | -0.00013 | 0.02 |
|  | Abscisic acid (ABA) | 265.1434 | 13.59 |  | 265.14334 | 13.57 | 13.45 - 13.76 |  |  |  | 0.00003 | 0.02 |
|  | Acacetin | 285.0752 | 16.65 |  | 285.07553 | 16.85 | 16.76 - 17.08 |  |  |  | -0.00030 | -0.2 |
|  | Adenine | 136.0615 | 1.64 |  | 136.06125 | 1.42 | 1.34 - 1.86 | yes |  |  | 0.00025 | 0.22 |
|  | Adenine | 136.0614 | 1.74 |  | 136.06125 | 1.77 | 1.34 - 1.86 | yes |  |  | 0.00015 | -0.03 |
|  | Adenine | 136.0615 | 1.58 |  | 136.06125 | 1.77 | 1.34 - 1.86 | yes |  |  | 0.00025 | -0.19 |
|  | Adenosine | 268.1034 | 1.8 |  | 268.10364 | 1.75 | 1.49 and 1.75 | yes |  |  | -0.00024 | 0.05 |
|  | Adenosine | 268.1035 | 1.52 |  | 268.10364 | 1.75 | 1.49 and 1.75 | yes |  |  | -0.00014 | -0.23 |
|  | Alanine | 90.0545 | 1.51 |  | 90.0546 | 1.43 | 1.36 - 1.52 |  |  |  | -0.00010 | 0.08 |
|  | Alanine | 90.05447 | 1.62 |  | 90.0546 | 1.43 | 1.36 - 1.52 |  |  |  | -0.00013 | 0.19 |
|  | Apigenin | 271.0596 | 14.63 |  | 271.05988 | 14.6 | 14.5 - 14.9 |  |  |  | -0.00028 | 0.03 |
|  | Arginine | 175.1187 | 1.38 |  | 175.119 | 1.34 | 1.26 - 1.52 |  |  |  | -0.00030 | 0.04 |
|  | Arginine | 175.1186 | 1.46 |  | 175.119 | 1.34 | 1.26 - 1.52 |  |  |  | -0.00040 | 0.12 |
|  | Asparagine | 133.0602 | 1.63 |  | 133.0607 | 1.46 | 1.37 - 1.57 |  |  |  | -0.00050 | 0.17 |
|  | Carvone | 151.1112 | 16.57 |  | 151.11151 | 16.76 | 16.63 - 17.08 |  |  |  | -0.00031 | -0.19 |
|  | Caryophyllene | 203.1793 | 21.49 |  | 203.1795 | 21.46 | 20.87 - 21.75 |  | yes |  | -0.00020 | 0.03 |
|  | Caryophyllene | 221.1895 | 21.51 |  | 221.1899 | 21.46 | 20.87 - 21.75 |  |  |  | -0.00040 | 0.05 |
|  | Caryophyllene | 203.179 | 21.08 |  | 203.1795 | 21.46 | 20.87 - 21.75 |  | yes |  | -0.00050 | -0.38 |
|  | Caryophyllene | 221.1894 | 21.08 |  | 221.1899 | 21.46 | 20.87 - 21.75 |  |  |  | -0.00050 | -0.38 |
|  | Catechin | 291.0876 | 3.44 |  | 291.08722 | 3.42 | 3.3 - 3.7 |  |  |  | 0.00038 | 0.02 |
|  | Catechin | 291.0877 | 3.3 |  | 291.08722 | 3.42 | 3.3 - 3.7 |  |  |  | 0.00048 | -0.12 |
|  | Catechin | 139.0385 | 3.44 |  | 139.03883 | 3.42 | 3.3 - 3.7 |  | yes |  | -0.00033 | 0.02 |
|  | Catechol (Pirocatequina) | 110.0196 | 4.03 |  | 110.01967 | 4.01 | 3.85 - 4.1 |  |  |  | -0.00007 | 0.02 |
|  | Catechol (Pirocatequina) | 110.0196 | 3.99 |  | 110.01967 | 4.01 | 3.85 - 4.1 |  |  |  | -0.00007 | -0.02 |
|  | Choline | 104.1066 | 1.43 |  | 104.10658 | 1.31 | 1.22 - 1.45 |  |  |  | 0.00002 | 0.12 |
|  | Choline | 104.1066 | 1.42 |  | 104.10658 | 1.31 | 1.22 - 1.45 |  |  |  | 0.00002 | 0.11 |
|  | Cytosine | 112.0503 | 1.36 |  | 112.05017 | 1.31 | 1.26 - 1.41 |  |  |  | 0.00013 | 0.05 |
|  | Eugenol | 163.0748 | 16.2 |  | 163.07529 | 15.92 | 15.75 - 17 |  |  |  | -0.00049 | 0.28 |
|  | Eugenol | 131.0486 | 16.2 |  | 131.04883 | 15.92 | 15.75 - 17 |  | yes |  | -0.00023 | 0.28 |
|  | Farnesol | 223.2052 | 19.23 |  | 223.2052 | 18.9 | 18.8 - 19.2 |  |  |  | 0.00000 | 0.33 |
|  | Farnesol | 205.195 | 19.25 |  | 205.1949 | 18.9 | 18.8 - 19.2 | yes |  |  | 0.00010 | 0.35 |
|  | Fisetin | 287.055 | 12.02 |  | 287.05521 | 12.41 | 12.25 - 13.66 |  |  |  | -0.00019 | -0.39 |
|  | Fisetin | 287.055 | 11.96 |  | 287.05521 | 12.41 | 12.25 - 13.66 |  |  |  | -0.00020 | -0.45 |
|  | Gibberellic acid (GA1) | 331.1537 | 11.01 |  | 331.15381 | 10.61 | 10.5 - 10.79 | yes |  |  | -0.00011 | 0.4 |
|  | Gibberellic acid (GA1) | 331.1535 | 11.08 |  | 331.15381 | 10.61 | 10.5 - 10.79 | yes |  |  | -0.00031 | 0.47 |
|  | Gibberellic acid (GA1) | 331.1535 | 10.46 |  | 331.15381 | 10.61 | 10.5 - 10.79 | yes |  |  | -0.00031 | -0.15 |
|  | Gibberellic acid (GA1) | 331.1535 | 10.92 |  | 331.15381 | 10.61 | 10.5 - 10.79 | yes |  |  | -0.00031 | 0.31 |
|  | Gibberellic acid (GA1) | 349.1635 | 10.46 |  | 349.16385 | 10.61 | 10.5 - 10.79 |  |  |  | -0.00035 | -0.15 |
|  | Glutamic acid | 148.0602 | 1.61 |  | 148.06035 | 1.43 | 1.35 - 1.6 |  |  |  | -0.00015 | 0.18 |
|  | Glutamic acid | 148.0602 | 1.52 |  | 148.06035 | 1.43 | 1.35 - 1.6 |  |  |  | -0.00015 | 0.09 |
|  | Glutamine | 147.0763 | 1.5 |  | 147.0763 | 1.46 | 1.37 - 1.63 |  |  |  | 0.00000 | 0.04 |
|  | Glutamine | 130.0495 | 1.55 |  | 130.049 | 1.46 | 1.37 - 1.63 |  | yes |  | 0.00050 | 0.09 |
|  | Glycine | 76.0388 | 1.63 |  | 76.039 | 1.43 | 1.35 - 1.54 |  |  |  | -0.00020 | 0.2 |
|  | Glycine | 76.03884 | 1.51 |  | 76.039 | 1.43 | 1.35 - 1.54 |  |  |  | -0.00016 | 0.08 |
|  | Guanine | 152.0562 | 1.45 |  | 152.05624 | 1.45 | 1.40 - 1.49 |  |  |  | -0.00004 | 0 |
|  | Isoleucine | 86.09596 | 1.52 |  | 86.096 | 1.7 | 1.52 - 1.77 |  | yes |  | -0.00004 | -0.18 |
|  | Isoleucine | 132.1016 | 1.52 |  | 132.1019 | 1.7 | 1.52 - 1.77 |  |  |  | -0.00030 | -0.18 |
|  | Isoleucine | 86.09597 | 1.82 |  | 86.096 | 1.7 | 1.52 - 1.77 |  | yes |  | -0.00003 | 0.12 |
|  | Isoleucine | 132.1016 | 1.82 |  | 132.1019 | 1.7 | 1.52 - 1.77 |  |  |  | -0.00030 | 0.12 |
|  | Lysine | 147.1123 | 1.59 |  | 147.1127 | 1.59 | 1.28 - 1.45 |  |  |  | -0.00040 | 0 |
|  | Methionine | 150.0579 | 1.53 |  | 150.058 | 1.59 | 1.48 - 1.71 |  |  |  | -0.00010 | -0.06 |
|  | Methionine | 150.058 | 1.75 |  | 150.058 | 1.59 | 1.48 - 1.71 |  |  |  | 0.00000 | 0.16 |
|  | Pantothenic acid (Vit B5) | 220.1177 | 2.01 |  | 220.11769 | 2 | 1.79 - 2.17 |  |  |  | 0.00001 | 0.01 |
|  | Phenylalanine | 166.0862 | 1.93 |  | 166.0864 | 1.91 | 1.83 - 2.07 |  |  |  | -0.00020 | 0.02 |
|  | Phenylalanine | 120.0804 | 1.93 |  | 120.08 | 1.91 | 1.83 - 2.07 |  | yes |  | 0.00040 | 0.02 |
|  | Proline | 70.0647 | 1.5 |  | 70.0647 | 1.49 | 1.38 - 1.60 |  | yes |  | 0.00000 | 0.01 |
|  | Proline | 116.0703 | 1.5 |  | 116.0703 | 1.49 | 1.38 - 1.60 |  |  |  | 0.00000 | 0.01 |
|  | Proline | 116.0702 | 1.63 |  | 116.0703 | 1.49 | 1.38 - 1.60 |  |  |  | -0.00010 | 0.14 |
|  | Robinetin | 303.0494 | 11.21 |  | 303.0498 | 10.75 | 10.49 - 12.5 |  |  |  | -0.00040 | 0.46 |
|  | Sabinene | 81.06935 | 21.87 |  | 81.0694 | 21.8 | 21.76 -22.14 | yes |  |  | -0.00005 | 0.07 |
|  | Sabinene | 137.1319 | 21.83 |  | 137.13199 | 21.8 | 21.76 -22.14 |  |  |  | -0.00009 | 0.03 |
|  | Serine | 106.0494 | 1.62 |  | 106.0495 | 1.47 | 1.41 - 1.58 |  |  |  | -0.00010 | 0.15 |
|  | Serine | 106.0494 | 1.53 |  | 106.0495 | 1.47 | 1.41 - 1.58 |  |  |  | -0.00010 | 0.06 |
|  | Threonine | 120.0651 | 1.51 |  | 120.065 | 1.43 | 1.38 - 1.64 |  |  |  | 0.00010 | 0.08 |
|  | Threonine | 120.065 | 1.64 |  | 120.065 | 1.43 | 1.38 - 1.64 |  |  |  | 0.00000 | 0.21 |
|  | Threonine | 102.0545 | 1.59 |  | 102.0547 | 1.43 | 1.38 - 1.64 |  | yes |  | -0.00020 | 0.16 |
|  | Thymine | 127.14983 | 1.97 |  | 127.04986 | 1.73 | 1.68 - 1.79 |  |  |  | -0.00002 | 0.24 |
|  | Tryptophan | 205.0972 | 2.51 |  | 205.097 | 2.49 | 2.40 - 2.70 |  |  |  | 0.00020 | 0.02 |
|  | Tryptophan | 188.0704 | 2.51 |  | 188.0708 | 2.49 | 2.40 - 2.70 |  | yes |  | -0.00040 | 0.02 |
|  | Tyrosine | 182.0809 | 1.53 |  | 182.0814 | 1.77 | 1.51 - 1.87 |  |  |  | -0.00050 | -0.24 |
|  | Tyrosine | 182.0809 | 1.76 |  | 182.0814 | 1.77 | 1.51 - 1.87 |  |  |  | -0.00050 | -0.01 |
|  | Uracil | 113.034 | 1.55 |  | 113.03407 | 1.49 | 1.45 - 1.79 |  |  |  | -0.00007 | 0.06 |
|  | Uridine | 245.0762 | 1.75 |  | 245.07669 | 1.76 | 1.42 - 1.83 |  |  |  | -0.00049 | -0.01 |
|  | Valine | 72.08035 | 1.51 |  | 72.0804 | 1.53 | 1.44 - 1.64 |  | yes |  | -0.00005 | -0.02 |
|  | Valine | 118.0859 | 1.51 |  | 118.0861 | 1.53 | 1.44 - 1.64 |  |  |  | -0.00020 | -0.02 |
|  | Valine | 118.0858 | 1.68 |  | 118.0861 | 1.53 | 1.44 - 1.64 |  |  |  | -0.00030 | 0.15 |
|  | Vitexin | 433.1121 | 11.11 |  | 433.11154 | 10.98 | 10.0 -11.5 |  |  |  | 0.00056 | 0.13 |

**Table S3.** Means ± SEs of the average maximum and minimum temperatures (°C) and the accumulated rainfalls (mm) for Navacerrada and Sierra Nevada Natural Park in winter and summer. Statistical *t* and *P* values comparing both localities for each variable are shown.

|  | Winter | | | |  | Summer | | | |
| --- | --- | --- | --- | --- | --- | --- | --- | --- | --- |
|  | Navacerrada | Sierra Nevada |  | |  | Navacerrada | Sierra Nevada |  | |
|  | Mean ± SE | Mean ± SE | *t* | *P* |  | Mean ± SE | Mean ± SE | *t* | *P* |
| Average maximum temperature (°C) | 5.99 ± 0.34 | 9.57 ± 0.41 | -6.65 | < 0.0001 |  | 22.99 ± 0.37 | 26.89 ± 0.47 | -0.58 | < 0.0001 |
| Average minimum temperature  (°C) | -1.14 ± 0.23 | -0.6 ± 0.26 | -1.27 | 0.21 |  | 11.38 ± 0.25 | 12.9 ± 0.3 | -3.88 | < 0.001 |
| Accumulated rainfall (mm) | 247.9 ± 28.3 | 272.4 ± 38.8 | 0.51 | 0.61 |  | 90.1 ± 9.4 | 54.3 ± 8.5 | -2.81 | < 0.01 |

**Table S4**. One-way ANOVAs for all known variables for each subspecies and season with folivory level (FL; NATs, AT.NABs, AT.ABs) as the categorical factor. Different letters denote statistical significance after HSD Tukey posthoc tests (P<0.05). P values are shown without and with Benjamini-Hochberg correction. Values for the metabolites represent the area values obtained after the deconvolution of the ion chromatograms.

| ***iberica* - winter** | | | | | | | | | | | |  |  |
| --- | --- | --- | --- | --- | --- | --- | --- | --- | --- | --- | --- | --- | --- |
|  | **NATs** | | | | **AT.NABs** | | | **AT.ABs** | | |  |  |  |
|  | **Mean** | **SE** |  | | **Mean** | **SE** |  | **Mean** | **SE** |  | **F** | **P** | **Corrected P values** |
| Sugars 1 (Deoxy-glucose - Deoxy-galactose - D-Fucose) | 3026451 | 317953 |  | | 2925341 | 298523 |  | 2521991 | 261629 |  | 0.83 | 0.44657 | 0.48717 |
| Disaccharides | 1749602 | 261486 | (b) | | 1674909 | 309784 | (b) | 9354318 | 926945 | (a) | 57.06 | 0.00000 | 0.00000 |
| Hexoses | 344485608 | 8352092 | (b) | | 377470808 | 9577468 | (a) | 287906637 | 8369363 | (c) | 26.59 | 0.00000 | 0.00000 |
| Pentoses | 26391103 | 1997070 |  | | 27268291 | 1163537 |  | 26512232 | 1429652 |  | 0.09 | 0.91254 | 0.91254 |
| Sugars 2 (Raffinose pentahydrate – Maltotriose) | 1518333 | 200706 | (b) | | 1126713 | 190737 | (b) | 2959719 | 353424 | (a) | 13.87 | 0.00004 | 0.00022 |
| Sugars 3 (Xylitol - Arabitol) | 62303465 | 4182704 | (b) | | 78969172 | 4022352 | (a) | 58172802 | 3173854 | (b) | 8.31 | 0.00119 | 0.00277 |
| Alanine | 25662770 | 3196983 | (a) | | 21470401 | 4109364 | (a) | 7144197 | 1508788 | (b) | 9.63 | 0.00051 | 0.00131 |
| Arginine | 2922139 | 457631 |  | | 1658608 | 267959 |  | 2808192 | 1093962 |  | 0.99 | 0.38178 | 0.42289 |
| Asparagine | 619434 | 117858 | (a) | | 378908 | 99890 | (a) | 35047 | 2977 | (b) | 10.84 | 0.00024 | 0.00083 |
| Aspartic acid | 1485651 | 132176 | (ab) | | 1832383 | 147261 | (a) | 1061679 | 90603 | (b) | 8.99 | 0.00080 | 0.00191 |
| Glutamic acid | 6689213 | 1276749 | (b) | | 6307236 | 535479 | (b) | 44389780 | 5665871 | (a) | 42.21 | 0.00000 | 0.00000 |
| Glutamine | 58996019 | 5541018 |  | | 69861562 | 12270915 |  | 60801282 | 8480715 |  | 0.40 | 0.67243 | 0.71198 |
| Glycine | 7537659 | 993139 | (a) | | 5951499 | 1089814 | (a) | 2099858 | 373176 | (b) | 10.14 | 0.00037 | 0.00102 |
| Isoleucine | 879948490 | 64203231 | (a) | | 728165352 | 75822674 | (a) | 244092117 | 38683245 | (b) | 29.10 | 0.00000 | 0.00000 |
| Leucine | 12144686 | 2156162 |  | | 9818560 | 1659282 |  | 7645150 | 549711 |  | 1.97 | 0.15529 | 0.18635 |
| Lysine | 2753876 | 350964 | (a) | | 2713551 | 443772 | (a) | 482094 | 79769 | (b) | 15.53 | 0.00002 | 0.00012 |
| Methionine | 87772957 | 6338844 | (a) | | 66889972 | 7959704 | (a) | 38798539 | 6850552 | (b) | 12.04 | 0.00012 | 0.00049 |
| Phenylalanine | 489158700 | 35897187 | (a) | | 448282239 | 43825114 | (a) | 160483288 | 23056762 | (b) | 25.73 | 0.00000 | 0.00000 |
| Proline | 57641156 | 7318584 | (a) | | 41614505 | 6936358 | (ab) | 34153217 | 6230622 | (b) | 3.08 | 0.05959 | 0.08757 |
| Serine | 22493203 | 2433032 | (a) | | 19003911 | 2862731 | (a) | 10372469 | 2043850 | (b) | 6.38 | 0.00453 | 0.00881 |
| Threonine | 27896517 | 3254165 | (a) | | 25210888 | 3467242 | (a) | 10571433 | 1282168 | (b) | 10.75 | 0.00025 | 0.00083 |
| Tryptophan | 367356805 | 47425633 |  | | 325821937 | 34086093 |  | 353957151 | 39621907 |  | 0.27 | 0.76455 | 0.78640 |
| Tyrosine | 53141629 | 5397037 | (a) | | 43795130 | 6360331 | (a) | 13886094 | 2054027 | (b) | 17.09 | 0.00001 | 0.00006 |
| Valine | 75896198 | 7173128 | (a) | | 61254024 | 10213763 | (a) | 30138248 | 4489446 | (b) | 9.31 | 0.00062 | 0.00154 |
| Adenine | 160397031 | 21163845 | (a) | | 144031314 | 19432332 | (ab) | 82323423 | 13517292 | (b) | 5.04 | 0.01226 | 0.02102 |
| Adenosine | 457786 | 74396 | (b) | | 368761 | 69896 | (b) | 1793804 | 427036 | (a) | 9.92 | 0.00042 | 0.00113 |
| Cytosine | 1140565 | 366348 |  | | 1424332 | 515298 |  | 367936 | 38219 |  | 2.24 | 0.12293 | 0.16093 |
| Guanine | 1416943 | 197135 | (a) | | 751179 | 156809 | (b) | 291730 | 47790 | (b) | 14.61 | 0.00003 | 0.00017 |
| Uridine | 596892 | 72263 |  | | 636785 | 92149 |  | 419277 | 64216 |  | 2.32 | 0.11465 | 0.15287 |
| Uracil | 571398 | 86659 | (a) | | 505381 | 76085 | (ab) | 339128 | 58411 | (b) | 2.57 | 0.09164 | 0.12937 |
| Citric acid | 247181996 | 12661205 | (a) | | 190348517 | 8997086 | (b) | 225238455 | 10854213 | (ab) | 6.86 | 0.00322 | 0.00644 |
| Lactic acid | 11202708 | 1392793 |  | | 12431376 | 1441763 |  | 10649735 | 1094546 |  | 0.48 | 0.62410 | 0.67067 |
| Malic acid | 506916808 | 30479367 | (b) | | 648455688 | 46089043 | (a) | 525023245 | 42972207 | (ab) | 3.63 | 0.03751 | 0.05627 |
| Oxaloacetic acid | 777067 | 48756 |  | | 810922 | 27829 |  | 764849 | 48336 |  | 0.31 | 0.73453 | 0.76647 |
| Pyruvic acid | 38768586 | 2698927 |  | | 38786067 | 4024588 |  | 30792140 | 2548229 |  | 2.13 | 0.13522 | 0.17385 |
| Succinic acid | 13770526 | 1083518 | (c) | | 17403515 | 843569 | (b) | 20843866 | 880799 | (a) | 14.10 | 0.00004 | 0.00021 |
| Vanillic acid | 3445505 | 216215 | (a) | | 3643886 | 581601 | (a) | 804301 | 203104 | (b) | 17.69 | 0.00001 | 0.00005 |
| Acacetin | 544097 | 103762 |  | | 1591905 | 559831 |  | 838565 | 199051 |  | 2.41 | 0.10563 | 0.14350 |
| Apigenin | 26445606 | 3012362 | (a) | | 32740092 | 2490281 | (a) | 14093958 | 2294349 | (b) | 13.14 | 0.00006 | 0.00030 |
| Caffeic acid | 5265560 | 734115 | (a) | | 4185475 | 996910 | (a) | 682217 | 165396 | (b) | 11.04 | 0.00021 | 0.00081 |
| Catechin | 221564952 | 23484289 | (b) | | 266674942 | 38509998 | (b) | 534965097 | 76325862 | (a) | 10.96 | 0.00022 | 0.00081 |
| Catechol | 25203987 | 1371666 | (b) | | 34486172 | 6503183 | (b) | 66447201 | 9611826 | (a) | 10.28 | 0.00034 | 0.00102 |
| Coumaric acid | 1050108 | 216159 | (a) | | 954766 | 135845 | (a) | 339745 | 72422 | (b) | 6.33 | 0.00470 | 0.00891 |
| D-Pinitol | 256555436 | 12201886 |  | | 277233062 | 13250008 |  | 254810425 | 10866524 |  | 1.05 | 0.35978 | 0.41118 |
| Epicatechin | 137810 | 28110 | (ab) | | 101383 | 12706 | (b) | 330546 | 93313 | (a) | 4.71 | 0.01587 | 0.02597 |
| Epigallocatechin | 32515596 | 1971538 | (b) | | 42602532 | 3937689 | (ab) | 47455955 | 3807482 | (a) | 5.14 | 0.01138 | 0.01998 |
| Ferulic acid | 6702323 | 899668 | (a) | | 6854024 | 1319361 | (a) | 1091568 | 227149 | (b) | 12.44 | 0.00009 | 0.00042 |
| Galagin | 5084 | 2033 |  | | 2479 | 663 |  | 2156 | 450 |  | 1.36 | 0.27153 | 0.31533 |
| Kaempferol | 4824215 | 522036 | (ab) | | 6202818 | 667734 | (a) | 3953231 | 558918 | (b) | 3.74 | 0.03423 | 0.05357 |
| Myricetin | 666851 | 153148 | (a) | | 620628 | 122821 | (a) | 109140 | 25826 | (b) | 7.75 | 0.00180 | 0.00381 |
| Quercitin | 2092134 | 194654 | (b) | | 3235431 | 308889 | (a) | 1368442 | 196844 | (b) | 15.45 | 0.00002 | 0.00012 |
| Quinic acid | 86993141 | 11129252 |  | | 118400511 | 15486205 |  | 121847378 | 13580368 |  | 2.02 | 0.14888 | 0.18169 |
| Resveratrol | 267430 | 53611 | (a) | | 142127 | 32998 | (ab) | 34720 | 4173 | (b) | 10.22 | 0.00035 | 0.00102 |
| Rhamnetin | 550873 | 46810 | (ab) | | 810846 | 121952 | (a) | 408711 | 68459 | (b) | 5.74 | 0.00728 | 0.01324 |
| Robinetin | 218905 | 28122 |  | | 396002 | 79215 |  | 363135 | 101053 |  | 1.52 | 0.23444 | 0.27672 |
| Sodium salicylate | 1866800 | 323981 | (ab) | | 1945620 | 443648 | (a) | 735103 | 156825 | (b) | 4.22 | 0.02340 | 0.03745 |
| Taxifolin | 33408205 | 1817716 |  | | 26588615 | 1646243 |  | 27108756 | 3896825 |  | 2.04 | 0.14621 | 0.18150 |
| Vitexin | 942988 | 198079 | (b) | | 1144456 | 160331 | (b) | 3656037 | 437647 | (a) | 26.73 | 0.00000 | 0.00000 |
| Flavone | 33202145 | 7225730 | (b) | | 107537879 | 27906686 | (a) | 53374327 | 8336171 | (ab) | 4.92 | 0.01346 | 0.02253 |
| Choline | 206573858 | 35106750 |  | | 143758867 | 25739641 |  | 128038386 | 15012091 |  | 2.44 | 0.10246 | 0.14186 |
| delta-tocopherol | 8773166 | 1168916 | (b) | | 9320882 | 1438916 | (b) | 30388775 | 2864831 | (a) | 39.14 | 0.00000 | 0.00000 |
| Eugenol | 3585333 | 994449 | (b) | | 42365208 | 8168438 | (a) | 54942176 | 14192726 | (a) | 7.99 | 0.00148 | 0.00323 |
| Vitamine B5 | 8317364 | 961721 | (a) | | 8876326 | 1362147 | (a) | 4374834 | 613043 | (b) | 5.72 | 0.00735 | 0.01324 |
| Alpha-ketoglutarate | 1824023 | 496914 | (a) | | 1127477 | 193404 | (ab) | 812560 | 124265 | (b) | 2.68 | 0.08338 | 0.12007 |
| Shikimic acid | 518181917 | 39641552 |  | | 537346543 | 25725733 |  | 465010939 | 43163478 |  | 1.03 | 0.36869 | 0.41478 |
| Sabinene | 3527984 | 524495 |  | | 3776249 | 692365 |  | 3237763 | 726849 |  | 0.17 | 0.84450 | 0.85639 |
| Carvone | 387443 | 123586 | (b) | | 586252 | 111800 | (b) | 3129965 | 746693 | (a) | 11.99 | 0.00012 | 0.00049 |
| Caryophyllene | 46986208 | 6971542 | (b) | | 66763943 | 9731486 | (ab) | 98674319 | 11192502 | (a) | 7.60 | 0.00193 | 0.00397 |
| Farnesol | 98626742 | 12954794 |  | | 94146647 | 9585884 |  | 69521356 | 9564876 |  | 2.10 | 0.13878 | 0.17530 |
| Absisic Acid | 4592533 | 505198 | (a) | | 5658721 | 948165 | (a) | 2028854 | 390660 | (b) | 7.99 | 0.00148 | 0.00323 |
| Gibberellic acid 1 | 8088628 | 930383 | (ab) | | 5299358 | 1031844 | (b) | 9838940 | 1518737 | (a) | 3.71 | 0.03516 | 0.05386 |
| Gibberellic acid 3 | 41379331 | 6300349 | (a) | | 32946541 | 5638354 | (a) | 10386376 | 2093395 | (b) | 10.15 | 0.00037 | 0.00102 |
|  | | | | | | | | | | | |  |  |
| ***nevadensis* - winter** | | | | | | | | | | | |  |  |
|  | **NATs** | | | **AT.NABs** | | | | **AT.ABs** | | |  |  |  |
|  | **Mean** | **SE** |  | **Mean** | | **SE** |  | **Mean** | **SE** |  | **P** | **F** | **Corrected P values** |
| Sugars 1 (Deoxy-glucose - Deoxy-galactose - D-Fucose) | 3578440 | 420732 |  | 4687322 | | 482966 |  | 3531888 | 387789 |  | 2.29 | 0.11723 | 0.16881 |
| Disaccharides | 1925720 | 252759 | (b) | 2739098 | | 400564 | (b) | 7558501 | 1333546 | (a) | 13.89 | 0.00004 | 0.00101 |
| Hexoses | 362920080 | 9360056 | (ab) | 371995778 | | 11270365 | (a) | 334836087 | 8643653 | (b) | 3.89 | 0.03038 | 0.07056 |
| Pentoses | 35418043 | 3082270 |  | 39505762 | | 2363330 |  | 33486326 | 2006956 |  | 1.48 | 0.24179 | 0.30015 |
| Sugars 2 (Raffinose pentahydrate – Maltotriose) | 1374734 | 259035 |  | 1943666 | | 260476 |  | 1767912 | 291629 |  | 1.16 | 0.32679 | 0.38572 |
| Sugars 3 (Xylitol - Arabitol) | 76083673 | 10035648 | (ab) | 95580698 | | 4990066 | (a) | 73862583 | 4934309 | (b) | 2.86 | 0.07175 | 0.12210 |
| Alanine | 27879311 | 4306800 | (b) | 46146462 | | 4806229 | (a) | 27338584 | 5625666 | (b) | 4.69 | 0.01610 | 0.04459 |
| Arginine | 7987146 | 1920152 | (b) | 28615818 | | 6762616 | (a) | 10103732 | 2515461 | (b) | 6.93 | 0.00307 | 0.01727 |
| Asparagine | 887292 | 210306 | (a) | 1157855 | | 261100 | (a) | 210993 | 37200 | (b) | 6.27 | 0.00492 | 0.02150 |
| Aspartic acid | 1232624 | 96094 | (b) | 1932622 | | 201331 | (a) | 1284081 | 127031 | (b) | 6.93 | 0.00307 | 0.01727 |
| Glutamic acid | 7317043 | 1075479 | (b) | 8990429 | | 1955354 | (b) | 44694664 | 8207105 | (a) | 18.49 | 0.00000 | 0.00016 |
| Glutamine | 72480476 | 7834817 | (b) | 118676917 | | 10644690 | (a) | 140285741 | 17804696 | (a) | 7.32 | 0.00234 | 0.01725 |
| Glycine | 9441755 | 1513508 | (ab) | 13404807 | | 1590748 | (a) | 5679596 | 702774 | (b) | 8.42 | 0.00111 | 0.01108 |
| Isoleucine | 816607877 | 79554795 | (ab) | 1069648487 | | 110885719 | (a) | 572830745 | 94774536 | (b) | 6.71 | 0.00360 | 0.01727 |
| Leucine | 12602112 | 2110016 |  | 11107912 | | 1672040 |  | 11423160 | 1973199 |  | 0.17 | 0.84688 | 0.87108 |
| Lysine | 3411724 | 624788 | (b) | 7217791 | | 1333770 | (a) | 1090547 | 158973 | (b) | 13.08 | 0.00007 | 0.00118 |
| Methionine | 85891774 | 5492021 | (ab) | 94951700 | | 6294286 | (a) | 67915037 | 5871917 | (b) | 5.45 | 0.00902 | 0.03122 |
| Phenylalanine | 564314225 | 53644919 | (ab) | 739569509 | | 80081625 | (a) | 439089623 | 71493984 | (b) | 4.75 | 0.01544 | 0.04447 |
| Proline | 43523526 | 5154949 | (b) | 92497034 | | 13499384 | (a) | 100219290 | 14515764 | (a) | 6.76 | 0.00346 | 0.01727 |
| Serine | 24277596 | 3384618 | (ab) | 32659961 | | 1943476 | (a) | 22481884 | 2839575 | (b) | 3.80 | 0.03270 | 0.07358 |
| Threonine | 28557141 | 3731724 |  | 37379789 | | 4113806 |  | 27652150 | 3709738 |  | 1.94 | 0.15944 | 0.21937 |
| Tryptophan | 611906130 | 69604834 | (b) | 891514098 | | 66374190 | (a) | 721373558 | 81857428 | (ab) | 3.73 | 0.03453 | 0.07535 |
| Tyrosine | 52886499 | 6580600 | (ab) | 69561864 | | 7983376 | (a) | 36494040 | 6449821 | (b) | 5.52 | 0.00857 | 0.03122 |
| Valine | 71020579 | 8670412 | (b) | 100768637 | | 9881441 | (a) | 73487231 | 10739423 | (ab) | 2.84 | 0.07292 | 0.12210 |
| Adenine | 166061591 | 11432677 |  | 201367002 | | 15333935 |  | 167113828 | 12585655 |  | 2.31 | 0.11520 | 0.16881 |
| Adenosine | 2543297 | 867874 | (ab) | 820952 | | 180604 | (b) | 2676141 | 585468 | (a) | 2.85 | 0.07235 | 0.12210 |
| Cytosine | 618940 | 189503 | (a) | 182320 | | 33300 | (b) | 153866 | 29117 | (b) | 5.38 | 0.00947 | 0.03122 |
| Guanine | 1068673 | 247903 | (ab) | 1786360 | | 349391 | (a) | 904947 | 198602 | (b) | 2.96 | 0.06586 | 0.12210 |
| Uridine | 657808 | 87940 |  | 809307 | | 104667 |  | 864098 | 96900 |  | 1.22 | 0.30821 | 0.36985 |
| Uracil | 683959 | 86993 | (b) | 800911 | | 119230 | (ab) | 1107969 | 98587 | (a) | 4.57 | 0.01774 | 0.04732 |
| Citric acid | 242025214 | 15205738 |  | 218839316 | | 10821115 |  | 232638057 | 10715845 |  | 0.88 | 0.42386 | 0.48298 |
| Lactic acid | 16204558 | 1317969 | (a) | 11693727 | | 1396313 | (b) | 9417723 | 1107495 | (b) | 7.29 | 0.00240 | 0.01725 |
| Malic acid | 544468503 | 25091895 | (a) | 446779744 | | 36928356 | (b) | 532369419 | 35984653 | (a) | 2.59 | 0.09041 | 0.14151 |
| Oxaloacetic acid | 815973 | 77051 |  | 897832 | | 58497 |  | 930911 | 64410 |  | 0.78 | 0.46776 | 0.51814 |
| Pyruvic acid | 47004966 | 5195912 |  | 37903754 | | 3394435 |  | 35277950 | 3262737 |  | 2.31 | 0.11499 | 0.16881 |
| Succinic acid | 16667453 | 683843 |  | 17341627 | | 746895 |  | 17235067 | 536720 |  | 0.30 | 0.74284 | 0.77514 |
| Vanillic acid | 2363606 | 429656 |  | 3204481 | | 539269 |  | 1958148 | 391732 |  | 1.93 | 0.16148 | 0.21937 |
| Acacetin | 502018 | 80325 |  | 924659 | | 284217 |  | 1303797 | 484959 |  | 1.50 | 0.23864 | 0.30015 |
| Apigenin | 48241508 | 4980447 |  | 51576222 | | 7090907 |  | 35614236 | 6962680 |  | 1.72 | 0.19452 | 0.25465 |
| Caffeic acid | 14125860 | 2968046 | (a) | 11695966 | | 2317732 | (ab) | 5162802 | 1249949 | (b) | 4.09 | 0.02580 | 0.06192 |
| Catechin | 307105568 | 28705637 | (b) | 325529390 | | 24913321 | (b) | 498675686 | 58371905 | (a) | 6.91 | 0.00312 | 0.01727 |
| Catechol | 59561071 | 7692309 |  | 54427721 | | 7255213 |  | 46586417 | 5985534 |  | 0.87 | 0.42932 | 0.48298 |
| Coumaric acid | 599564 | 95988 |  | 675327 | | 96683 |  | 465304 | 84813 |  | 1.32 | 0.28146 | 0.34348 |
| D-Pinitol | 286920685 | 17889287 | (a) | 261264188 | | 18574937 | (ab) | 230757056 | 13009836 | (b) | 2.84 | 0.07261 | 0.12210 |
| Epicatechin | 138141 | 26286 | (ab) | 110139 | | 15555 | (b) | 231137 | 59607 | (a) | 2.68 | 0.08324 | 0.13621 |
| Epigallocatechin | 37222276 | 2966753 |  | 31974534 | | 2827846 |  | 33281511 | 2769526 |  | 0.91 | 0.41045 | 0.47665 |
| Ferulic acid | 5862046 | 910735 | (a) | 5989206 | | 989285 | (a) | 2595817 | 423177 | (b) | 5.59 | 0.00814 | 0.03122 |
| Galagin | 6122 | 1590 |  | 9179 | | 2974 |  | 8393 | 2208 |  | 0.48 | 0.62161 | 0.66800 |
| Kaempferol | 6489855 | 661179 |  | 7043838 | | 705731 |  | 5839215 | 967950 |  | 0.58 | 0.56410 | 0.61538 |
| Myricetin | 868418 | 133822 | (a) | 533875 | | 130745 | (ab) | 363953 | 57082 | (b) | 5.17 | 0.01118 | 0.03499 |
| Quercitin | 3859156 | 653242 | (a) | 4445357 | | 384323 | (a) | 2300750 | 132175 | (b) | 6.23 | 0.00508 | 0.02150 |
| Quinic acid | 97008760 | 11358206 |  | 97934606 | | 11490684 |  | 104455927 | 12224290 |  | 0.12 | 0.88695 | 0.88695 |
| Resveratrol | 351586 | 60987 | (a) | 206240 | | 46146 | (ab) | 90041 | 18625 | (b) | 8.31 | 0.00119 | 0.01108 |
| Rhamnetin | 685072 | 100115 |  | 989081 | | 151462 |  | 769100 | 116048 |  | 1.59 | 0.21867 | 0.28115 |
| Robinetin | 770575 | 162661 | (b) | 1808222 | | 333281 | (a) | 891826 | 205248 | (b) | 5.37 | 0.00954 | 0.03122 |
| Sodium salicylate | 2778005 | 363353 | (a) | 2120828 | | 383685 | (ab) | 1481252 | 399573 | (b) | 2.87 | 0.07071 | 0.12210 |
| Taxifolin | 32952071 | 3310512 |  | 34844078 | | 4504494 |  | 35862041 | 3630220 |  | 0.15 | 0.86365 | 0.87581 |
| Vitexin | 2184306 | 511895 | (b) | 2109530 | | 363386 | (b) | 4211985 | 919609 | (a) | 3.44 | 0.04384 | 0.08768 |
| Flavone | 68292522 | 12337846 | (b) | 133188875 | | 21505945 | (a) | 79761959 | 14922147 | (ab) | 4.30 | 0.02194 | 0.05642 |
| Choline | 197475987 | 29897211 | (b) | 303498222 | | 36212170 | (a) | 270092978 | 23119308 | (ab) | 3.22 | 0.05290 | 0.10293 |
| delta-tocopherol | 9147077 | 941241 | (b) | 11624151 | | 1079361 | (b) | 22132265 | 3372710 | (a) | 10.62 | 0.00027 | 0.00396 |
| Eugenol | 9155366 | 2735959 | (b) | 15012029 | | 3382254 | (b) | 60072852 | 16212413 | (a) | 8.26 | 0.00123 | 0.01108 |
| Vitamine B5 | 12820552 | 1575122 |  | 17861041 | | 1952745 |  | 14360995 | 1521817 |  | 2.32 | 0.11364 | 0.16881 |
| Alpha-ketoglutarate | 2032827 | 318028 | (a) | 1737833 | | 392683 | (ab) | 1059034 | 175937 | (b) | 2.61 | 0.08844 | 0.14150 |
| Shikimic acid | 518242036 | 23782932 | (ab) | 535687962 | | 23055010 | (a) | 436775594 | 29839853 | (b) | 4.21 | 0.02357 | 0.05853 |
| Sabinene | 6332029 | 1361598 |  | 5913177 | | 1162725 |  | 3396073 | 700810 |  | 2.05 | 0.14528 | 0.20510 |
| Carvone | 406231 | 63919 | (b) | 848237 | | 207040 | (b) | 4784626 | 952112 | (a) | 18.28 | 0.00000 | 0.00016 |
| Caryophyllene | 92128972 | 12164854 |  | 118772028 | | 19715510 |  | 140598860 | 21176713 |  | 1.79 | 0.18206 | 0.24274 |
| Farnesol | 144490982 | 26159807 | (a) | 119834635 | | 20136693 | (ab) | 69756127 | 10445775 | (b) | 3.63 | 0.03763 | 0.07741 |
| Absisic Acid | 10505634 | 1160868 | (ab) | 13260675 | | 2023837 | (a) | 7554433 | 1122768 | (b) | 3.64 | 0.03716 | 0.07741 |
| Gibberellic acid 1 | 25751594 | 3336524 |  | 28254188 | | 1664499 |  | 28783953 | 3425072 |  | 0.31 | 0.73774 | 0.77514 |
| Gibberellic acid 3 | 44184754 | 6182680 | (a) | 35448577 | | 6084816 | (ab) | 21356939 | 2469186 | (b) | 4.89 | 0.01378 | 0.04133 |
|  | | | | | | | | | | | |  |  |
| ***iberica* - summer** | | | | | | | | | | | |  |  |
|  | **NATs** | | | **AT.NABs** | | | | **AT.ABs** | | |  |  |  |
|  | **Mean** | **SE** |  | **Mean** | | **SE** |  | **Mean** | **SE** |  | **F** | **P** | **Corrected P values** |
| Sugars 1 (Deoxy-glucose - Deoxy-galactose - D-Fucose) | 2480887 | 196346 | (a) | 2621782 | | 269833 | (a) | 1419450 | 231132 | (b) | 7.87 | 0.00161 | 0.00809 |
| Disaccharides | 6079 | 1362 | (b) | 7675 | | 2059 | (b) | 113606 | 33847 | (a) | 4.69 | 0.02387 | 0.05927 |
| Hexoses | 176254880 | 11890783 | (ab) | 194388904 | | 10661503 | (a) | 142687486 | 13774507 | (b) | 4.64 | 0.01675 | 0.04472 |
| Pentoses | 11925348 | 1123963 |  | 11865516 | | 991120 |  | 10174040 | 1000875 |  | 0.91 | 0.41109 | 0.53025 |
| Sugars 2 (Raffinose pentahydrate – Maltotriose) | 480264 | 52850 | (a) | 437490 | | 68063 | (a) | 232493 | 25695 | (b) | 6.51 | 0.00415 | 0.01658 |
| Sugars 3 (Xylitol - Arabitol) | 16536377 | 1340406 |  | 15841043 | | 1272199 |  | 16876247 | 1753541 |  | 0.13 | 0.87967 | 0.91792 |
| Alanine | 21087378 | 3190116 | (a) | 11280379 | | 1246735 | (b) | 9955919 | 1569461 | (b) | 7.81 | 0.00167 | 0.00809 |
| Arginine | 1259317 | 354573 | (a) | 233144 | | 49278 | (b) | 199320 | 51811 | (b) | 8.32 | 0.00118 | 0.00809 |
| Asparagine | 89634 | 24202 | (a) | 49437 | | 8254 | (ab) | 40486 | 8771 | (b) | 2.81 | 0.07447 | 0.14110 |
| Aspartic acid | 993994 | 111089 | (a) | 663648 | | 101318 | (b) | 660021 | 72706 | (b) | 3.96 | 0.02884 | 0.06698 |
| Glutamic acid | 192915193 | 21531553 | (a) | 129380891 | | 17658427 | (b) | 133385692 | 16146029 | (b) | 3.67 | 0.03650 | 0.08213 |
| Glutamine | 97930930 | 15757493 | (a) | 36845366 | | 3993038 | (b) | 36293005 | 5435197 | (b) | 12.82 | 0.00008 | 0.00182 |
| Glycine | 659289 | 86342 | (a) | 542425 | | 80826 | (ab) | 329130 | 56181 | (b) | 4.90 | 0.01365 | 0.04096 |
| Isoleucine | 567956008 | 106157304 | (a) | 293045218 | | 66401671 | (b) | 166248131 | 36456234 | (b) | 7.44 | 0.00215 | 0.00970 |
| Leucine | 8668598 | 959651 |  | 9248882 | | 2133931 |  | 7328892 | 791526 |  | 0.48 | 0.62498 | 0.73768 |
| Lysine | 917250 | 199442 |  | 726297 | | 144583 |  | 546116 | 151332 |  | 1.24 | 0.30356 | 0.42855 |
| Methionine | 34478601 | 5561495 |  | 24846323 | | 2181580 |  | 24472606 | 2438731 |  | 2.32 | 0.11428 | 0.17887 |
| Phenylalanine | 351975057 | 60602673 | (a) | 176920540 | | 35334214 | (b) | 119988797 | 21555301 | (b) | 8.14 | 0.00134 | 0.00809 |
| Proline | 29637017 | 4277490 | (a) | 18710640 | | 1956892 | (b) | 21755089 | 3374411 | (ab) | 2.85 | 0.07238 | 0.14084 |
| Serine | 24621498 | 2576494 | (a) | 15342143 | | 2134723 | (b) | 13001900 | 1649912 | (b) | 8.14 | 0.00134 | 0.00809 |
| Threonine | 24615782 | 2947284 | (a) | 15268420 | | 1820722 | (b) | 17930524 | 2728547 | (ab) | 3.58 | 0.03923 | 0.08560 |
| Tryptophan | 41047825 | 7262435 |  | 47135996 | | 7619235 |  | 54906397 | 12457821 |  | 0.54 | 0.58543 | 0.70251 |
| Tyrosine | 34239565 | 6628669 | (a) | 19671698 | | 3661342 | (ab) | 12345271 | 2051485 | (b) | 6.05 | 0.00576 | 0.01975 |
| Valine | 43168811 | 6704495 | (a) | 30379741 | | 5476802 | (ab) | 20392882 | 3289661 | (b) | 4.56 | 0.01785 | 0.04591 |
| Adenine | 69224403 | 8971697 |  | 59826548 | | 6702264 |  | 60526749 | 8148466 |  | 0.43 | 0.65493 | 0.74849 |
| Adenosine | 7500409 | 3418746 |  | 4230054 | | 1210235 |  | 7797005 | 2204087 |  | 0.65 | 0.52729 | 0.65457 |
| Cytosine | 150707 | 23839 |  | 136666 | | 22153 |  | 107818 | 8462 |  | 1.27 | 0.29456 | 0.42417 |
| Guanine | 288043 | 49551 |  | 190716 | | 38392 |  | 179964 | 21498 |  | 2.42 | 0.10438 | 0.17494 |
| Uridine | 1206340 | 269708 |  | 1111186 | | 85956 |  | 1178162 | 107834 |  | 0.08 | 0.92504 | 0.95147 |
| Uracil | 1188503 | 307376 |  | 1230846 | | 128134 |  | 1173158 | 135734 |  | 0.02 | 0.97952 | 0.97952 |
| Citric acid | 101054634 | 3897460 | (a) | 85728928 | | 6876053 | (ab) | 68400028 | 5414127 | (b) | 8.72 | 0.00091 | 0.00809 |
| Lactic acid | 11951253 | 780835 |  | 11812076 | | 690331 |  | 12122101 | 1023427 |  | 0.03 | 0.96670 | 0.97952 |
| Malic acid | 162542504 | 3023504 | (b) | 166697291 | | 9800291 | (b) | 229736756 | 20476125 | (a) | 8.11 | 0.00137 | 0.00809 |
| Oxaloacetic acid | 2522147 | 280736 | (a) | 2209809 | | 168911 | (a) | 1342174 | 142811 | (b) | 8.78 | 0.00088 | 0.00809 |
| Pyruvic acid | 39449392 | 5224677 |  | 32316779 | | 2082746 |  | 37748435 | 6495847 |  | 0.56 | 0.57436 | 0.70091 |
| Succinic acid | 26962702 | 719528 | (a) | 24921091 | | 972226 | (a) | 15175648 | 1503063 | (b) | 31.98 | 0.00000 | 0.00000 |
| Vanillic acid | 475736 | 121180 |  | 672805 | | 174631 |  | 421622 | 111528 |  | 0.91 | 0.41242 | 0.53025 |
| Acacetin | 58674 | 15567 | (b) | 319127 | | 121587 | (ab) | 495861 | 143492 | (a) | 4.07 | 0.02622 | 0.06294 |
| Apigenin | 1791412 | 495185 |  | 674242 | | 208758 |  | 1034688 | 331077 |  | 2.45 | 0.10201 | 0.17494 |
| Caffeic acid | 789516 | 171860 |  | 678671 | | 211475 |  | 309404 | 70138 |  | 2.39 | 0.10691 | 0.17494 |
| Catechin | 53426051 | 6612636 | (b) | 79083061 | | 6383932 | (a) | 94046081 | 8801694 | (a) | 7.82 | 0.00166 | 0.00809 |
| Catechol | 9421234 | 1211565 |  | 10225627 | | 926096 |  | 9834233 | 821828 |  | 0.16 | 0.85132 | 0.90140 |
| Coumaric acid | 787434 | 166787 | (a) | 296737 | | 57002 | (b) | 312460 | 77813 | (b) | 6.29 | 0.00487 | 0.01844 |
| D-Pinitol | 232655733 | 12211280 | (a) | 203104430 | | 14306899 | (ab) | 182309884 | 14791839 | (b) | 3.35 | 0.04723 | 0.09715 |
| Epicatechin | 4059 | 1270 |  | 4343 | | 1137 |  | 4932 | 742 |  | 0.18 | 0.84024 | 0.90140 |
| Epigallocatechin | 19875087 | 2753397 | (b) | 36973431 | | 3519773 | (a) | 31471559 | 3056491 | (a) | 7.80 | 0.00169 | 0.00809 |
| Ferulic acid | 9254507 | 1783784 | (a) | 4786858 | | 1121188 | (ab) | 3049049 | 756510 | (b) | 6.13 | 0.00543 | 0.01954 |
| Galagin | 3024 | 371 |  | 2487 | | 470 |  | 2992 | 568 |  | 0.28 | 0.76126 | 0.84286 |
| Kaempferol | 7673485 | 904172 | (a) | 3737771 | | 680592 | (b) | 3572092 | 766673 | (b) | 8.65 | 0.00095 | 0.00809 |
| Myricetin | 1079258 | 186542 | (a) | 499844 | | 112660 | (b) | 694773 | 177656 | (ab) | 3.30 | 0.04943 | 0.09885 |
| Quercitin | 1165496 | 180134 | (a) | 962009 | | 165287 | (a) | 442023 | 58475 | (b) | 6.61 | 0.00386 | 0.01633 |
| Quinic acid | 1958624368 | 137724185 | (ab) | 2134221206 | | 122067918 | (a) | 1669643912 | 115513098 | (b) | 3.50 | 0.04194 | 0.08882 |
| Resveratrol | 3078 | 519 |  | 2235 | | 414 |  | 3183 | 1135 |  | 0.35 | 0.70695 | 0.79531 |
| Rhamnetin | 1825142 | 209010 | (a) | 787086 | | 113777 | (b) | 550648 | 109859 | (b) | 20.07 | 0.00000 | 0.00007 |
| Robinetin | 64613 | 11541 |  | 93161 | | 19736 |  | 113508 | 20607 |  | 1.70 | 0.20006 | 0.29397 |
| Sodium salicylate | 1545668 | 283039 |  | 1135811 | | 162417 |  | 887938 | 175939 |  | 2.41 | 0.10561 | 0.17494 |
| Taxifolin | 7860143 | 1418587 | (a) | 5188357 | | 795985 | (ab) | 3611026 | 533912 | (b) | 4.72 | 0.01572 | 0.04472 |
| Vitexin | 4693410 | 606751 | (b) | 10466401 | | 1765764 | (a) | 10572596 | 1485505 | (a) | 5.96 | 0.00615 | 0.02013 |
| Flavone | 1326390 | 376773 | (b) | 2554073 | | 355363 | (ab) | 4432087 | 990290 | (a) | 5.88 | 0.00656 | 0.02053 |
| Choline | 107005825 | 17408607 |  | 87385125 | | 13788386 |  | 79718786 | 18964151 |  | 0.70 | 0.50540 | 0.63840 |
| delta-tocopherol | 4095442 | 365841 |  | 5943468 | | 1063520 |  | 5587555 | 1099193 |  | 1.17 | 0.32406 | 0.44870 |
| Eugenol | 6902 | 1657 | (b) | 4260 | | 630 | (b) | 16623 | 2967 | (a) | 10.65 | 0.00027 | 0.00487 |
| Vitamine B5 | 23411419 | 2426922 |  | 19026320 | | 1484688 |  | 18742551 | 1818943 |  | 1.80 | 0.18074 | 0.27687 |
| Alpha-ketoglutarate | 658466 | 61006 |  | 739408 | | 165940 |  | 413017 | 64592 |  | 2.45 | 0.10219 | 0.17494 |
| Shikimic acid | 542312641 | 24158457 | (a) | 555989367 | | 23566173 | (a) | 438901216 | 38876724 | (b) | 4.64 | 0.01677 | 0.04472 |
| Sabinene | 1417291 | 268141 |  | 2116691 | | 408419 |  | 1692901 | 337019 |  | 1.06 | 0.35898 | 0.47863 |
| Carvone | 47180 | 10516 |  | 45608 | | 9353 |  | 63898 | 8900 |  | 1.11 | 0.34102 | 0.46328 |
| Caryophyllene | 13946355 | 2276525 |  | 16644848 | | 1619199 |  | 20930005 | 2847559 |  | 2.34 | 0.11229 | 0.17887 |
| Farnesol | 81068508 | 11835618 |  | 81919976 | | 16826882 |  | 100067244 | 18879124 |  | 0.44 | 0.64578 | 0.74849 |
| Absisic Acid | 2891021 | 482254 |  | 3253689 | | 616029 |  | 1974740 | 378543 |  | 1.73 | 0.19376 | 0.29063 |
| Gibberellic acid 1 | 34642587 | 2095433 |  | 32280194 | | 2177737 |  | 33380283 | 2644111 |  | 0.26 | 0.77262 | 0.84286 |
| Gibberellic acid 3 | 41332269 | 7183543 | (a) | 29702975 | | 4417442 | (ab) | 24109564 | 4566235 | (b) | 2.52 | 0.09600 | 0.17494 |
|  | | | | | | | | | | | |  |  |
| ***nevadensis* - summer** | | | | | | | | | | | |  |  |
|  | **NATs** | | | **AT.NABs** | | | | **AT.ABs** | | |  |  |  |
|  | **MEAN** | **SE** |  | **MEAN** | | **SE** |  | **MEAN** | **SE** |  | **F** | **P** | **Corrected P values** |
| Sugars 1 (Deoxy-glucose - Deoxy-galactose - D-Fucose) | 1054320 | 66513 |  | 1196262 | | 111280 |  | 1168104 | 169152 |  | 0.37 | 0.69150 | 0.71759 |
| Disaccharides | 1285088 | 284405 | (b) | 2592647 | | 639046 | (ab) | 4324440 | 838406 | (a) | 5.85 | 0.00669 | 0.05685 |
| Hexoses | 119442969 | 10051954 |  | 144197889 | | 12505947 |  | 117283931 | 10643798 |  | 1.81 | 0.17957 | 0.36941 |
| Pentoses | 9937782 | 991384 |  | 8554754 | | 532847 |  | 10956173 | 974715 |  | 1.97 | 0.15607 | 0.33175 |
| Sugars 2 (Raffinose pentahydrate – Maltotriose) | 264842 | 38028 | (a) | 173420 | | 13736 | (b) | 193257 | 19588 | (ab) | 3.44 | 0.04406 | 0.18205 |
| Sugars 3 (Xylitol - Arabitol) | 17895572 | 1205045 |  | 20882516 | | 1481660 |  | 20433419 | 1693813 |  | 1.19 | 0.31569 | 0.47354 |
| Alanine | 17171429 | 1512783 |  | 15355160 | | 2436826 |  | 13613013 | 1860785 |  | 0.81 | 0.45240 | 0.57059 |
| Arginine | 4823185 | 1564390 | (a) | 874053 | | 293239 | (b) | 1027408 | 271313 | (b) | 5.76 | 0.00715 | 0.05685 |
| Asparagine | 88251 | 15046 |  | 62776 | | 14035 |  | 58743 | 17477 |  | 1.05 | 0.36008 | 0.51851 |
| Aspartic acid | 753463 | 120550 | (a) | 566176 | | 35637 | (ab) | 484603 | 71102 | (b) | 2.73 | 0.07973 | 0.23920 |
| Glutamic acid | 197766242 | 16914970 |  | 177292426 | | 16692196 |  | 167770468 | 18470678 |  | 0.78 | 0.46757 | 0.57059 |
| Glutamine | 117421823 | 18957679 | (a) | 64934172 | | 6985773 | (b) | 59594430 | 11675135 | (b) | 5.63 | 0.00790 | 0.05685 |
| Glycine | 497034 | 83335 | (a) | 290577 | | 54211 | (b) | 240237 | 25529 | (b) | 5.27 | 0.01030 | 0.06742 |
| Isoleucine | 153270331 | 18311671 | (ab) | 159600614 | | 26152890 | (a) | 98923257 | 8891846 | (b) | 3.04 | 0.06147 | 0.20117 |
| Leucine | 4629828 | 840298 |  | 5525138 | | 683358 |  | 5900982 | 496741 |  | 0.90 | 0.41590 | 0.55454 |
| Lysine | 259824 | 30534 |  | 285470 | | 52930 |  | 190239 | 31767 |  | 1.54 | 0.23027 | 0.41922 |
| Methionine | 33463049 | 2809048 | (a) | 35601569 | | 1246014 | (a) | 18144096 | 1633123 | (b) | 22.46 | 0.00000 | 0.00005 |
| Phenylalanine | 124290210 | 10482535 | (a) | 122906341 | | 18297025 | (a) | 82299248 | 8488524 | (b) | 3.30 | 0.04923 | 0.18654 |
| Proline | 18216409 | 2016065 |  | 14278974 | | 1409936 |  | 13582361 | 1480898 |  | 2.27 | 0.11903 | 0.31409 |
| Serine | 23368016 | 2276304 |  | 19389561 | | 2378578 |  | 16638077 | 2115962 |  | 2.24 | 0.12215 | 0.31409 |
| Threonine | 26415288 | 2431606 | (a) | 16395818 | | 1743194 | (b) | 17881280 | 2287098 | (b) | 6.18 | 0.00523 | 0.05384 |
| Tryptophan | 42487373 | 11205544 |  | 36101748 | | 6148043 |  | 29543963 | 2343843 |  | 0.74 | 0.48294 | 0.57613 |
| Tyrosine | 11341494 | 1200133 |  | 12138686 | | 1486218 |  | 10255309 | 1130721 |  | 0.54 | 0.58547 | 0.63870 |
| Valine | 20769882 | 1801700 |  | 17677971 | | 2141378 |  | 14776529 | 2182474 |  | 2.14 | 0.13373 | 0.32096 |
| Adenine | 60250606 | 7025307 |  | 60404108 | | 8079096 |  | 49359787 | 7591964 |  | 0.70 | 0.50459 | 0.58598 |
| Adenosine | 36805470 | 10303211 |  | 38127352 | | 8295962 |  | 46725000 | 8533421 |  | 0.35 | 0.70643 | 0.71759 |
| Cytosine | 146666 | 19676 |  | 110276 | | 24669 |  | 103747 | 8776 |  | 1.50 | 0.23890 | 0.41922 |
| Guanine | 258312 | 51277 |  | 317910 | | 47344 |  | 223901 | 32042 |  | 1.15 | 0.32876 | 0.48307 |
| Uridine | 2365322 | 350364 | (a) | 2681922 | | 345038 | (a) | 1144943 | 181336 | (b) | 7.19 | 0.00255 | 0.03677 |
| Uracil | 2068731 | 234691 | (ab) | 2607992 | | 315942 | (a) | 1202286 | 195946 | (b) | 7.81 | 0.00168 | 0.03017 |
| Citric acid | 81996658 | 6680209 | (a) | 65735048 | | 2473600 | (b) | 80000066 | 6657324 | (a) | 2.48 | 0.09904 | 0.27426 |
| Lactic acid | 8800859 | 894528 | (b) | 12455936 | | 744858 | (a) | 11813612 | 564262 | (a) | 6.83 | 0.00330 | 0.03962 |
| Malic acid | 267770508 | 16083565 |  | 270933857 | | 7664104 |  | 249716120 | 10179328 |  | 0.93 | 0.40327 | 0.55097 |
| Oxaloacetic acid | 1419748 | 206617 |  | 1519791 | | 121922 |  | 1370401 | 215458 |  | 0.17 | 0.84678 | 0.84678 |
| Pyruvic acid | 28884395 | 2139474 | (ab) | 23809883 | | 1498413 | (b) | 40789452 | 6636595 | (a) | 4.48 | 0.01899 | 0.09769 |
| Succinic acid | 12843573 | 495009 | (b) | 15162335 | | 654339 | (a) | 17033251 | 609801 | (a) | 12.65 | 0.00008 | 0.00302 |
| Vanillic acid | 243891 | 39153 |  | 183583 | | 22654 |  | 199097 | 60087 |  | 0.52 | 0.59924 | 0.64396 |
| Acacetin | 38486 | 8693 | (b) | 118915 | | 33589 | (a) | 83616 | 25340 | (ab) | 2.64 | 0.08629 | 0.24851 |
| Apigenin | 443545 | 110508 | (a) | 170118 | | 44042 | (b) | 267075 | 65339 | (ab) | 3.13 | 0.05694 | 0.19522 |
| Caffeic acid | 262710 | 58348 | (a) | 151900 | | 23824 | (ab) | 126801 | 31723 | (b) | 3.15 | 0.05590 | 0.19522 |
| Catechin | 100339862 | 9504257 |  | 111907705 | | 14759043 |  | 96382724 | 5056285 |  | 0.59 | 0.56275 | 0.63309 |
| Catechol | 9820570 | 743349 |  | 10163765 | | 1403648 |  | 8907116 | 1048172 |  | 0.35 | 0.70762 | 0.71759 |
| Coumaric acid | 122990 | 17217 |  | 145168 | | 15007 |  | 111561 | 21973 |  | 0.87 | 0.42750 | 0.55964 |
| D-Pinitol | 157880162 | 9380095 |  | 173439016 | | 10808111 |  | 171017205 | 13399796 |  | 0.55 | 0.58381 | 0.63870 |
| Epicatechin | 6838 | 1291 |  | 7346 | | 1524 |  | 4662 | 660 |  | 1.28 | 0.29083 | 0.45521 |
| Epigallocatechin | 24177948 | 2905689 |  | 18947523 | | 2082705 |  | 19789841 | 2612525 |  | 1.21 | 0.31200 | 0.47354 |
| Ferulic acid | 1364264 | 180284 |  | 1487081 | | 210556 |  | 1053666 | 147252 |  | 1.52 | 0.23373 | 0.41922 |
| Galagin | 1496 | 449 |  | 6749 | | 3082 |  | 3891 | 1632 |  | 2.20 | 0.13986 | 0.32483 |
| Kaempferol | 2733124 | 451935 |  | 2136943 | | 428105 |  | 2080051 | 188978 |  | 0.93 | 0.40557 | 0.55097 |
| Myricetin | 292187 | 72172 |  | 251586 | | 50645 |  | 188441 | 52037 |  | 0.78 | 0.46567 | 0.57059 |
| Quercitin | 312193 | 58985 |  | 198405 | | 42460 |  | 306504 | 63557 |  | 1.32 | 0.28008 | 0.44813 |
| Quinic acid | 1763555526 | 100855333 | (b) | 2150023264 | | 117413015 | (a) | 2041593110 | 105474299 | (ab) | 3.40 | 0.04551 | 0.18205 |
| Resveratrol | 2541 | 453 |  | 2048 | | 209 |  | 1714 | 348 |  | 1.42 | 0.26205 | 0.43878 |
| Rhamnetin | 528321 | 82575 |  | 473270 | | 69442 |  | 369988 | 51704 |  | 1.35 | 0.27214 | 0.44532 |
| Robinetin | 51465 | 9386 |  | 69314 | | 14855 |  | 66579 | 7204 |  | 0.73 | 0.48811 | 0.57613 |
| Sodium salicylate | 379115 | 59003 |  | 576225 | | 86340 |  | 424167 | 73325 |  | 1.96 | 0.15666 | 0.33175 |
| Taxifolin | 3280750 | 720775 |  | 4488453 | | 886342 |  | 3293850 | 484431 |  | 0.94 | 0.40195 | 0.55097 |
| Vitexin | 6084565 | 800012 |  | 7584229 | | 1351909 |  | 6868397 | 1270956 |  | 0.41 | 0.66478 | 0.70388 |
| Flavone | 2360084 | 603694 |  | 1716286 | | 224856 |  | 2393770 | 318343 |  | 0.85 | 0.43785 | 0.56295 |
| Choline | 149136316 | 19914050 |  | 101496325 | | 13942730 |  | 105612770 | 19601911 |  | 2.14 | 0.13328 | 0.32096 |
| delta-tocopherol | 7923482 | 1239402 |  | 7523061 | | 922092 |  | 10250372 | 1294281 |  | 1.60 | 0.21677 | 0.41922 |
| Eugenol | 9029 | 1554 | (b) | 16056 | | 4500 | (b) | 215673 | 69462 | (a) | 8.52 | 0.00104 | 0.02496 |
| Vitamine B5 | 22283914 | 2205378 |  | 20196601 | | 1253063 |  | 17513469 | 2216257 |  | 1.51 | 0.23531 | 0.41922 |
| Alpha-ketoglutarate | 459970 | 94611 | (a) | 168077 | | 19968 | (b) | 296987 | 64714 | (ab) | 4.74 | 0.01548 | 0.09290 |
| Shikimic acid | 327319408 | 21225896 | (b) | 399730643 | | 30134785 | (ab) | 428501927 | 29897772 | (a) | 3.62 | 0.03789 | 0.17052 |
| Sabinene | 1330935 | 201913 | (ab) | 1018776 | | 205832 | (b) | 2062869 | 462865 | (a) | 2.90 | 0.06929 | 0.21692 |
| Carvone | 30424 | 7264 |  | 29395 | | 5878 |  | 53888 | 14194 |  | 1.99 | 0.15224 | 0.33175 |
| Caryophyllene | 22908058 | 2340636 |  | 19114476 | | 2286501 |  | 17990348 | 1685837 |  | 1.47 | 0.24455 | 0.41922 |
| Farnesol | 79566961 | 12261160 |  | 81790132 | | 15819417 |  | 62071476 | 11618801 |  | 0.65 | 0.52689 | 0.60217 |
| Absisic Acid | 1518065 | 316793 |  | 1368230 | | 141352 |  | 940289 | 210232 |  | 1.64 | 0.20955 | 0.41910 |
| Gibberellic acid 1 | 58237709 | 6691135 | (a) | 49896131 | | 3360037 | (ab) | 37766020 | 3677108 | (b) | 4.57 | 0.01772 | 0.09769 |
| Gibberellic acid 3 | 15839785 | 2594212 | (a) | 15294714 | | 2454543 | (a) | 8563824 | 824385 | (b) | 3.67 | 0.03645 | 0.17052 |

**Figure S1.** Representation of regions where needles were selected for the not-attacked trees (NATs), the non-attacked branches of the attacked trees (AT.NABs) and the attacked branches of the attacked trees (AT.ABs). All sampled needles were exposed to the sun during sampling. The tree image was drawn by Albert Rivas-Ubach with Adobe Photoshop CS6.

**
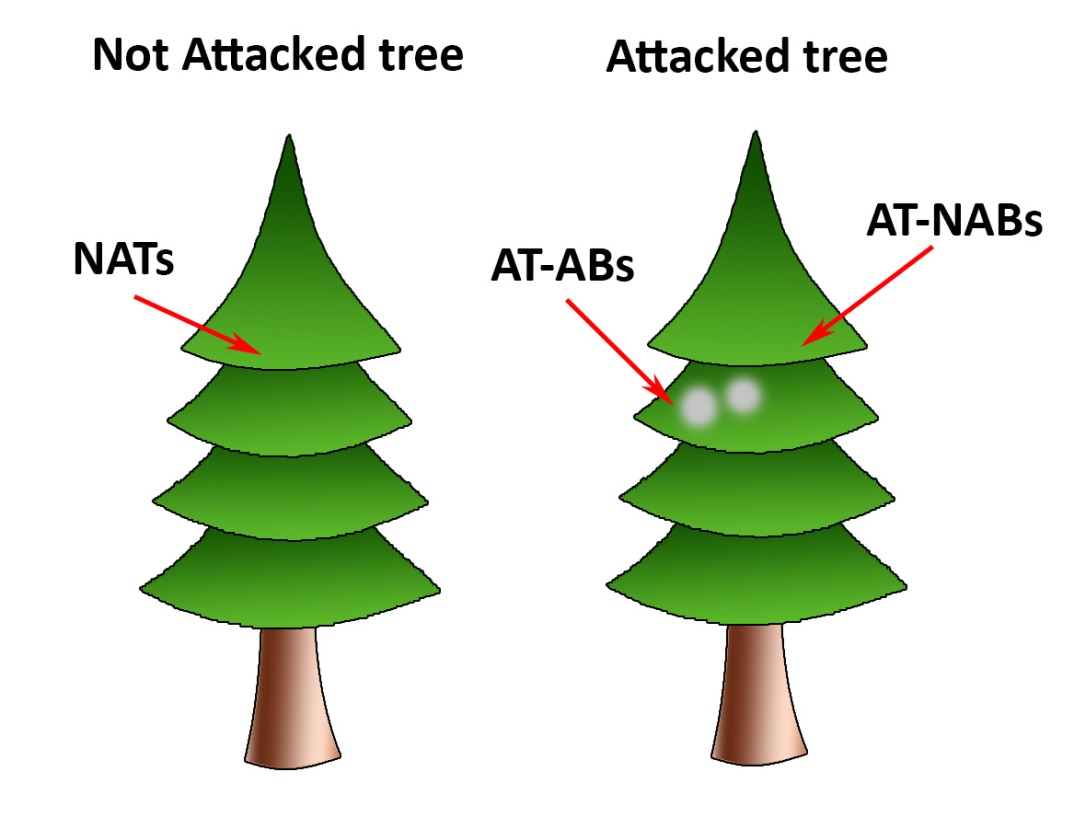
**

**Figure S2.** Schematic representation of the different Euclidean distance (black lines) combinations that were calculated in the study. Each combination of Eculidean distances was calculated for each season separately. The square represents the multivariate space composed by all metabolomic variables and the position of each subject (circles for *iberica* and crosses for *nevadensis*) would represent the metabolomic structure of that specific tree. Only two trees from each folivory level (FL) and subspecies were represented in the schemas. Different colors denote different FL (blue for NATS, green for AT.NABs and red for AT.ABs). Panels **a**, **b** and **c** represent the Euclidean distances between *iberica* and *nevadensis* for each FL (NATs *iberica* vs. NATs *nevadensis* (**a**), AT.NABs *iberica* vs. AT.NABs *nevadensis* (**b**) and AT.ABs *iberica* vs. AT.ABs *nevadensis* (**c**)). Panels **d** and **e** represent the Euclidean distances between FLs for each subspecies (NATs vs. AT.NABs vs. AT.ABs for *iberica* (**d**) and for *nevadensis* (**e**)).


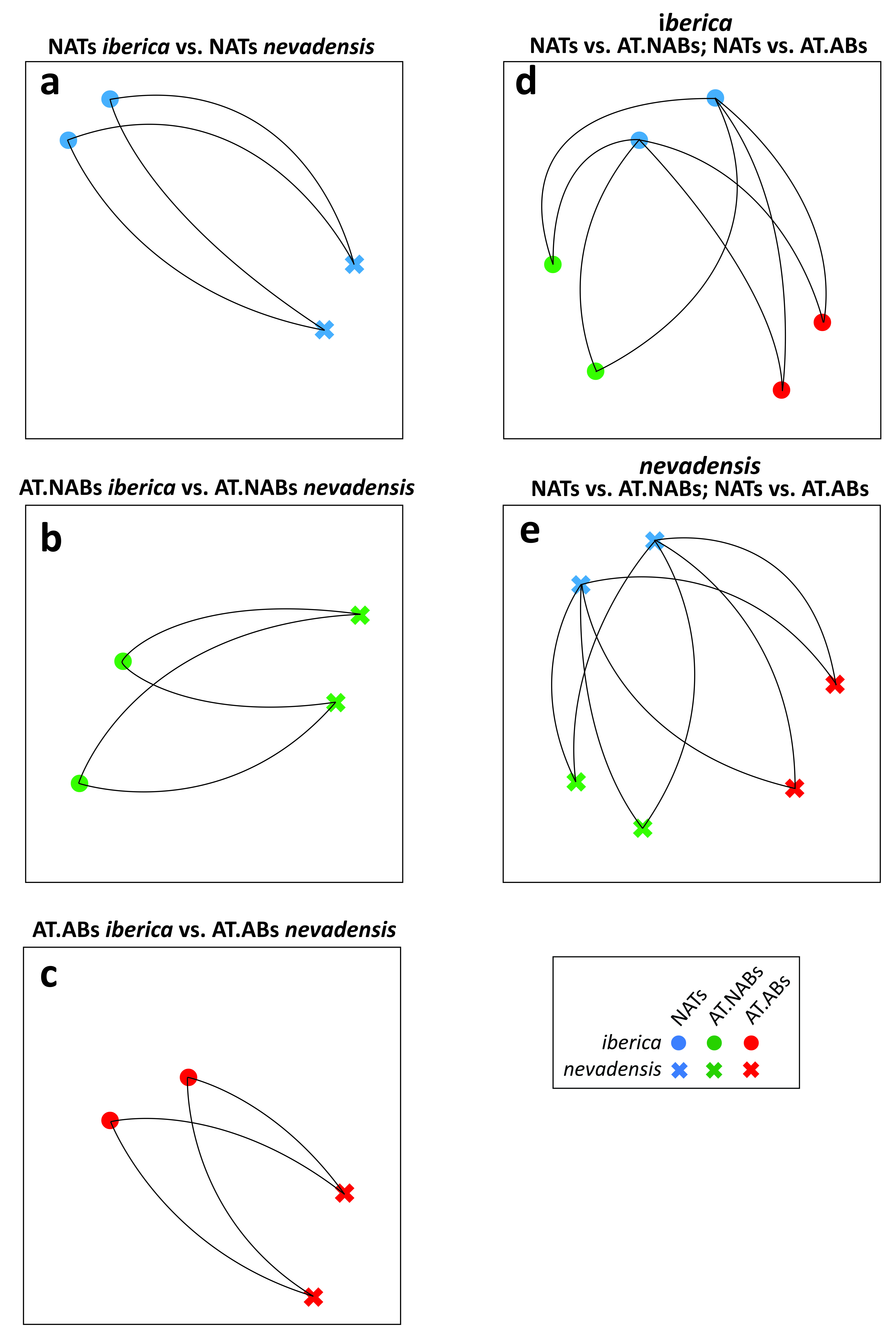

Supplement: Supplementary file 1 [file ECE3-7-8976-s001.docx]
